# Beyond-Kasha Photochemistry in a Heteroleptic Platinum–Dithiolene Complex

**DOI:** 10.1021/jacs.6c00565

**Published:** 2026-03-13

**Authors:** Michela Gazzetto, Flavia Artizzu, Salahuddin. S. Attar, Jakob T. Casanova, Luciano Marchiò, Luca Pilia, Antonio Monari, Paola Deplano, Andrea Cannizzo

**Affiliations:** † Institute of Applied Physics, 27210 University of Bern, Bern CH-3012, Switzerland; ‡ Department of Sustainable Development and Ecological Transition (DISSTE), 19050 University of Eastern Piedmont, Vercelli I-13100, Italy; § Dipartimento di Scienze Chimiche e Geologiche, Università di Cagliari, Monserrato (CA), Cagliari I-09042, Italy; ∥ Dipartimento di Scienze Chimiche, della Vita e della Sostenibilità Ambientale, Università di Parma, Parma I-43124, Italy; ⊥ Dipartimento di Ingegneria Meccanica, Chimica e dei Materiali, Università di Cagliari, Cagliari I-09123, Italy; # ITODYS, Université Paris Cité and CNRS, Paris F-75006, France

## Abstract

Kasha’s rule implies that photochemical reactions occur in the lowest excited state, regardless of excitation wavelength. Only a few chromophores have been reported to exhibit efficient non-Kasha responses. While these are rare, their exploitation could revolutionize multiresponsive materials, improve solar energy utilization, and advance light-driven chemical reactions. Studying non-Kasha dynamics enhances the understanding of excited-state processes and could broaden the range of usable chromophores in real applications. This article focuses on the anionic heteroleptic dithiolene complex [Pt­((R)-α-MBAdto)­(quinoxdt)]^−^ ((*R*)-α-MBAdto = (*R*)-(+)­α-methylbenzyl-dithiooxamidate; quinoxdt = [1,4]­dithiino­[2,3-*b*]­quinoxaline-2,3-bis­(thiolate)) in acetonitrile, which undergoes long-lived conformational changes exclusively upon excitation of higher excited states. In its tight ion-pair adduct with HCl, these changes drive HCl detachment within 70 ps, trigger a dramatic blue-shift in the ***S***_**1**_–***S***_**0**_ gap, and lead to aggregate formation. Although these processes ultimately occur in the lowest excited state, they rely on non-Kasha isomerization, representing a “beyond-Kasha” process. Such systems pave the way for innovative multiresponsive materials and non-Kasha excitation-dependent photochemical applications.

## Graphical abstract

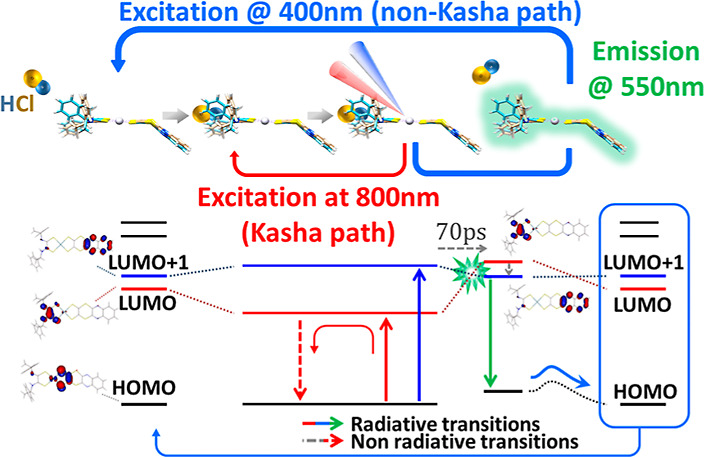

## Introduction

Kasha’s rule states, when adapted to photochemistry, that photochemical reactions in the condensed phase occur appreciably only in the lowest excited state of a given multiplicity, irrespective of the excitation wavelength (λ_Exc_). It affects any aspect of photochemistry and sets severe limitations to the efficiency of solar energy usage, e.g., for photocatalysis, or to controlling, not just triggering, chemical reactions using light. It also opposes the development of multiresponsive materials, whose light response could depend on the λ_Exc_ or on the number of excitations. Some of its violations are known^1,2^ and are extremely interesting for applications in the domain of multiresponsive photoactive materials,^3^ dual-emission probes,^2,4^ or efficient photon-energy utilization.^5^ Moreover, studying molecules showing a non-Kasha (nK), also called anti-Kasha,^2,4^ behavior can allow investigating relaxation mechanisms and molecular processes on, or from, higher excited states, benefiting the comprehension of fundamental molecular phenomena and the improvement of computational quantum chemistry approaches.^6^ Thanks to ultrafast spectroscopies and advances in excited-state computational methods, further evidence of the Kasha’s rule violation has been reported for the major types of excited-state reactions as photoisomerization, bond-breaking, and charge and energy transfers, raising a growing interest in nK photochemistry.^2,7^ However, only a few classes of chromophores, as azulene and their derivatives, Zn porphyrins, and some metal complexes,^2^ feature an nK response efficiently exploitable in real applications. A huge potential could be unleashed by increasing the classes of chromophores for nK applications.

Recently, a nK behavior was observed in d^8^-metal dithiolene complexes (MCs), both homoleptic^8^ and heteroleptic,^9,10^ containing the quinoxdt ([1,4]­dithiino­[2,3-*b*]­quinoxaline-2,3-bis­(thiolate)) ligand where the quinoxaline ring (quinox) is connected to the dithiolate C2S2 moiety through a 1,4-dithiine bridge.

These MCs^8,9^ in solution have been investigated with ultrafast spectroscopy,^11,12^ and an outstandingly long lifetime (1–2 ps) of the second singlet excited state (***S***_**2**_),^11^ comparable with the higher excited states lifetime in Zn porphyrins, was observed. This uncommon condition allows these MCs to emit detectable nK emission. Such an exceptionally slow internal conversion (IC) was rationalized as due to the fact that the first and second excited states have profoundly different electronic density distributions, with no overlap between the orbitals involved in the IC process (see Figure 1B and S1C). This different electron density localization makes the IC a long-range charge transfer (CT) process between the quinoxdt and the other ligand, characterized by a large reorganization energy. This condition dramatically slows the IC down.^10^

**Figure 1:**
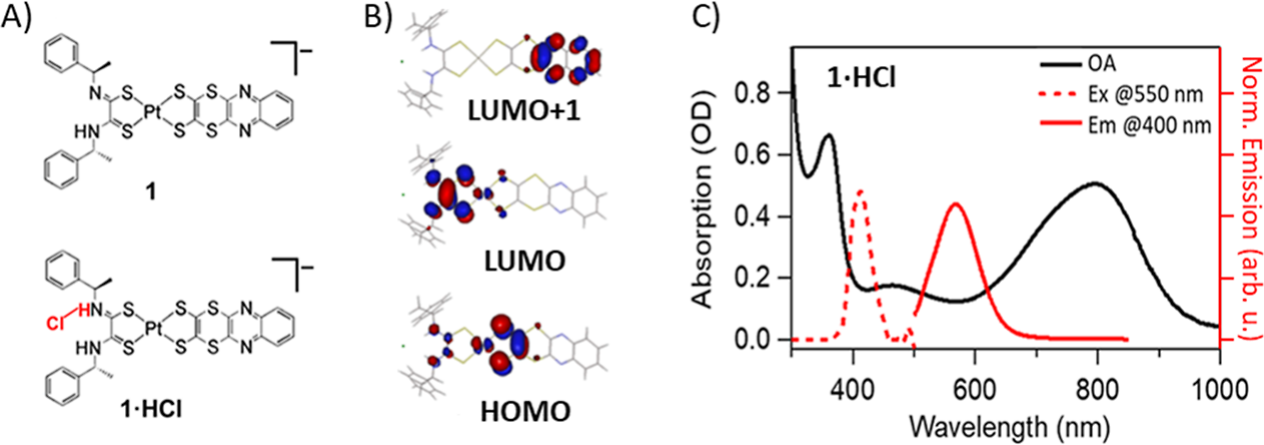
(A) Structural formulas of the precursor (**1**), namely, the parent molecule before adding HCl, and its HCl adduct (**1·HCl**). (B) DFT calculated molecular orbitals of **1·HCl**. (C) Steady-state absorption (OA, black line), non-Kasha emission excited at 400 nm (Em@400 nm, red solid line), and an excitation spectrum detected at 550 nm (Ex@550 nm, red dashed line) of **1·HCl** in acetonitrile. See the Supporting Information, Section SI.2, for more details.

Remarkably, the heteroleptic complex [Pt­((*R*)-α-MBAdto)­(quinoxdt)]^−^ (**1**), where (*R*)-α-MBAdto = (*R*)-(+)­α-methylbenzyl-dithiooxamidate (Figure 1A), undergoes photoisomerization, which is triggered only upon excitation of ***S***_**2**_ and, after the IC process toward the first singlet excited state (***S***_**1**_), continues to evolve. This process shows a high quantum yield (QY), is reversible, and is long-lived (at least ns).^11^

By HCl addition, **1** forms a 1:1 adduct (**1·HCl**, Figure 1A), which still preserves the nK behavior, despite its optical properties are dramatically changed.^8,9^ This motivated us to investigate also the nK dynamics of **1·HCl** with ultrafast transient absorption (TA) spectroscopy to understand whether and how the nK isomerization is affected by the **1·HCl** formation and to verify whether the CT character of the IC still applies. Eventually, these results may help to design new molecules with an enhanced nK emission QY.

The results reported in this study reveal that only the nK excitation can induce the breaking of the adduct with the release of HCl in tens of ps. We rationalize this observation as an effect of nK photoisomerization, which triggers a destabilization of **1·HCl** in acetonitrile. All the molecules undergo this reaction, as proved by the strong TA signals related to this process. Therefore, the system herein investigated can be considered the archetype of a new family of efficient multiresponsive materials which exploit the nK photoisomerization to achieve an excitation-dependent photochemical response. These results represent a proof of concept for new strategies based on selective triggering of photoisomerization, exploitable in the emerging field of nK photochemistry, where, so far, the dominant approach relies on increasing the higher excited-state lifetime to make the functional process competitive.^2^

## Results and Discussion

All the measurement in this article are carried out in acetonitrile. Figure 1C shows the steady-state UV–vis optical absorption, emission (λ_EXC_ = 400 nm), and excitation (λ_Em_ = 550 nm) spectra of **1·HCl**. The nK 550 nm emission^10^ is observed only upon HCl addition and with low QY (≲10^–3^), whereas no emission is observed exciting the lowest absorption band at 800 nm. The latter speaks for a strongly quenched fluorescence with a subμs lifetime.

As aforesaid, the S_2_ → S_1_ IC in **1** is astonishingly slow (∼1 ps against typically ∼10 fs)^11^ because the IC is a CT process associated with a remarkable change in the electronic distribution between the LUMO + 1 and the LUMO orbitals (Figure S1).^9^ This unusual condition makes the S_2_ emission detectable.

Before investigating the nK dynamics of **1·HCl**, we characterized the relaxations upon excitation of the lowest electronic transition (Figure 2A). We observe the same behavior (see the Supporting Information for details) of **1** upon excitation of ***S***_**1**_,^11^ namely: a 0.9 ps vibrational cooling followed by a 3.9 ps intersystem crossing (ISC) toward long-lived low-lying triplet states. This implies that the **1·HCl** formation has no effect on the ***S***_**1**_ dynamics. It is worth noting that both **1·HCl** and **1** do not show any emission upon excitation of the lowest absorption band.

**Figure 2:**
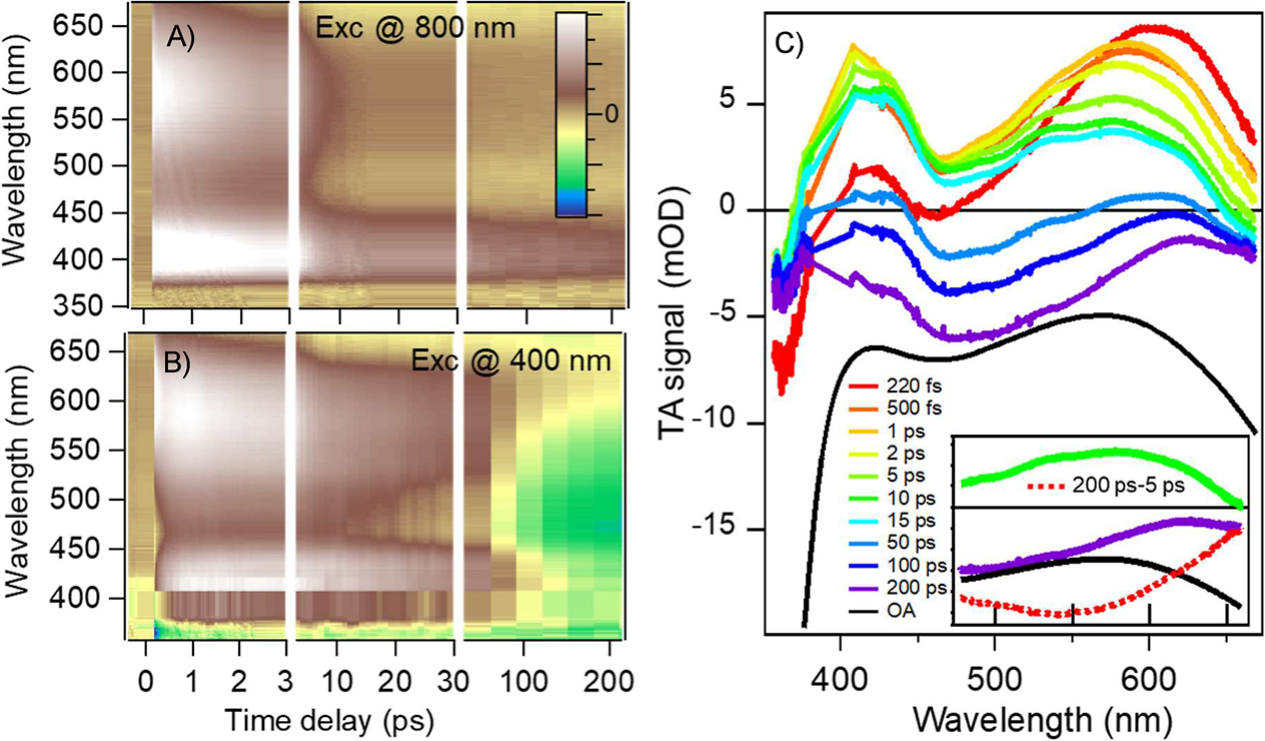
Transient absorption (TA) measurements upon (A) excitation at λ_Exc_ = 800 nm (B) and 400 nm. (C) Representative selection of TA spectra at different time delays from panel B. For the sake of comparison, the steady-state absorption band is shown inverted and suitably normalized. In the inset are shown the difference spectra between the TA spectra at 5 ps (green) and 200 ps (violet) to isolate the spectrum of the emission at 550 nm (dashed red line).

Conversely, adding HCl profoundly changes the TA signal (Figure 2B,C) upon excitation of the nK transition at 400 nm. The most striking result is the formation of a negative signal after 50 ps between 450 and 600 nm, which sits on top of the positive excited-state absorption (ESA) signals present from the very beginning. To isolate this contribution, we subtracted from a long-lived spectrum (200 ps) an early time spectrum (5 ps), revealing a negative signal centered at 550 nm (the red dashed line in the inset of Figure 2C). We chose 5 ps because it is late enough to assume vibrational relaxation and cooling dynamics completed but early enough to exclude contamination from the negative signal. The comparison of this isolated contribution with the steady-state emission in Figure 1C confirms that this signal is due to the stimulated emission (SE) corresponding to the nK emission. This emission is fully allowed since its amplitude is comparable with the ground-state bleach (GSB) one, and at least ns lived, lasting well beyond the investigated temporal window (up to 450 ps). This result is unexpected and somehow astonishing.

It is unexpected because a fully allowed (i.e., an expected radiative time of tens of ns) and long-lived emission (i.e., a total lifetime between few ns and the radiative time) should have an emission QY at least of 10^–1^, whereas the reported value^9^ is <10^–3^. It is astonishing because this allowed emission (therefore stemming from a singlet state) develops on a so long-time scale, ∼70 ps, that all the electronic and vibrational relaxation can be considered concluded, and λ_Exc_ should no longer play any role. However, upon direct excitation of ***S***_**1**_ at 800 nm, no emission is observed (Figure 2A).

To rationalize such unpredictable behavior, we need first to identify the electronic state populated after the 400 nm excitation and before the emission rise. The comparison with the TA signals excited at 800 nm (Figure S5) reveals that ESA and GSB bands excited at 400 and 800 nm in the first 10 ps are practically the same, showing that after the subpicosecond dynamics, **1·HCl** reaches the same excited state. However, upon 400 nm excitation, we observe a subpicosecond rise absent in the signal excited at 800 nm (Figure S6), which shows a more complex and faster picosecond evolution than the 400 nm one (Figure S7). Accordingly, we can conclude that upon 400 nm excitation, we populate the same electronic state excited at 800 nm, i.e., ***S***_**1**_, after an IC process lasting ∼1 ps (see the central part of Figure 3 for the photocycle describing the firsts ps). As for **1**,^11^ where a 1.4 ps *S*_2_ → *S*_1_ IC was reported, we explain it as an effect of the LUMO–LUMO + 1 spatial separation (Figure 3). However, since in **1·HCl** nK emission rises in 10 s of ps and lasts ns, it cannot originate from higher excited states lasting few ps.

**Figure 3:**
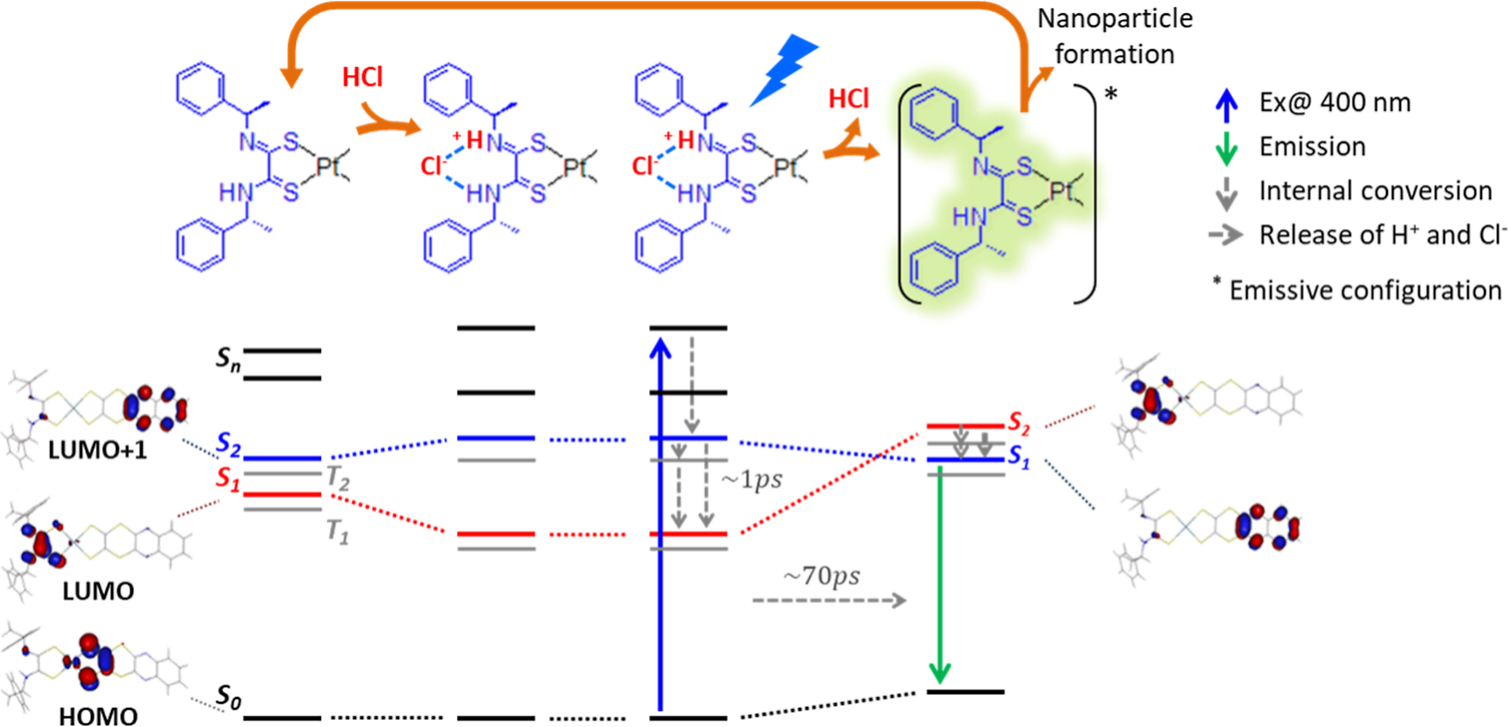
Full photocycle triggered by the 400 nm excitation and the effect of the HCl addition on the energetics (bottom). The top row shows the corresponding structural configurations. The relaxation toward the lowest excited state *S*_1_ involves a long-lived (∼1 ps) higher excited state *S*_2_. Upon HCl release in ∼70 ps (69 ± 3 ps from Figure S14), the system relaxes into an emissive configuration where the excited electron density is centered on the quinoxdt ligand (Figure S12 and relative discussion), from which it can relax back to the ground state or trigger aggregation. Both the 1 ps and the 70 ps relaxations are accompanied by conformational dynamics (a tentative conformational change is shown in Figure S12). To show the excited electron distribution, the involved molecular orbitals are drawn. For the sake of completeness also, the lowest triple states are shown; however, since the transient configurations are unknown, their relative position is tentative. Molecular orbitals and structures adapted from ref 9, copyright 2017 American Chemical Society.

A convincing explanation requires reconsidering how the emission QY, which is <10^–3^,^9,10^ was estimated. As aforesaid, results in Figure 2 contradict such a low value, implying QY > 10^–1^ and speaking for a quenching process in steady-state measurements, absent in a time-resolved experiment. The striking difference is that in steady-state measurements, a cuvette without circulation is used, and the molecules are excited multiple times. Conversely, the latter uses circulated solutions, with pump fluence and sample volume chosen to excite only a few percent of molecules during acquisition. To verify whether this is the cause, we ran consecutive TA experiments on a small volume (2 mL of solution with concentration *c* = 4.43 × 10^–4^ M) to reduce the solution buffer effect and with faster acquisition parameters (7 min for each experiment) to monitor possible photodegradation processes (Figure 4). Consistent with the first experiment (Figure 2), we initially observed the same emission. After 40 min, this emission is nearly fully quenched (inset A, Figure 4) but with a sigmoidal (inset B, Figure 4) rather than monoexponential decay, as expected for one photon degradation processes. Remarkably, after the SE quenching kinetics are completed, a negative signal, ∼100 times smaller than SE in the fresh sample, is observed exclusively upon 400 nm excitation (Figure S8). This implies a 10^–3^ emission QY, in excellent agreement with the literature.^9,10^

**Figure 4:**
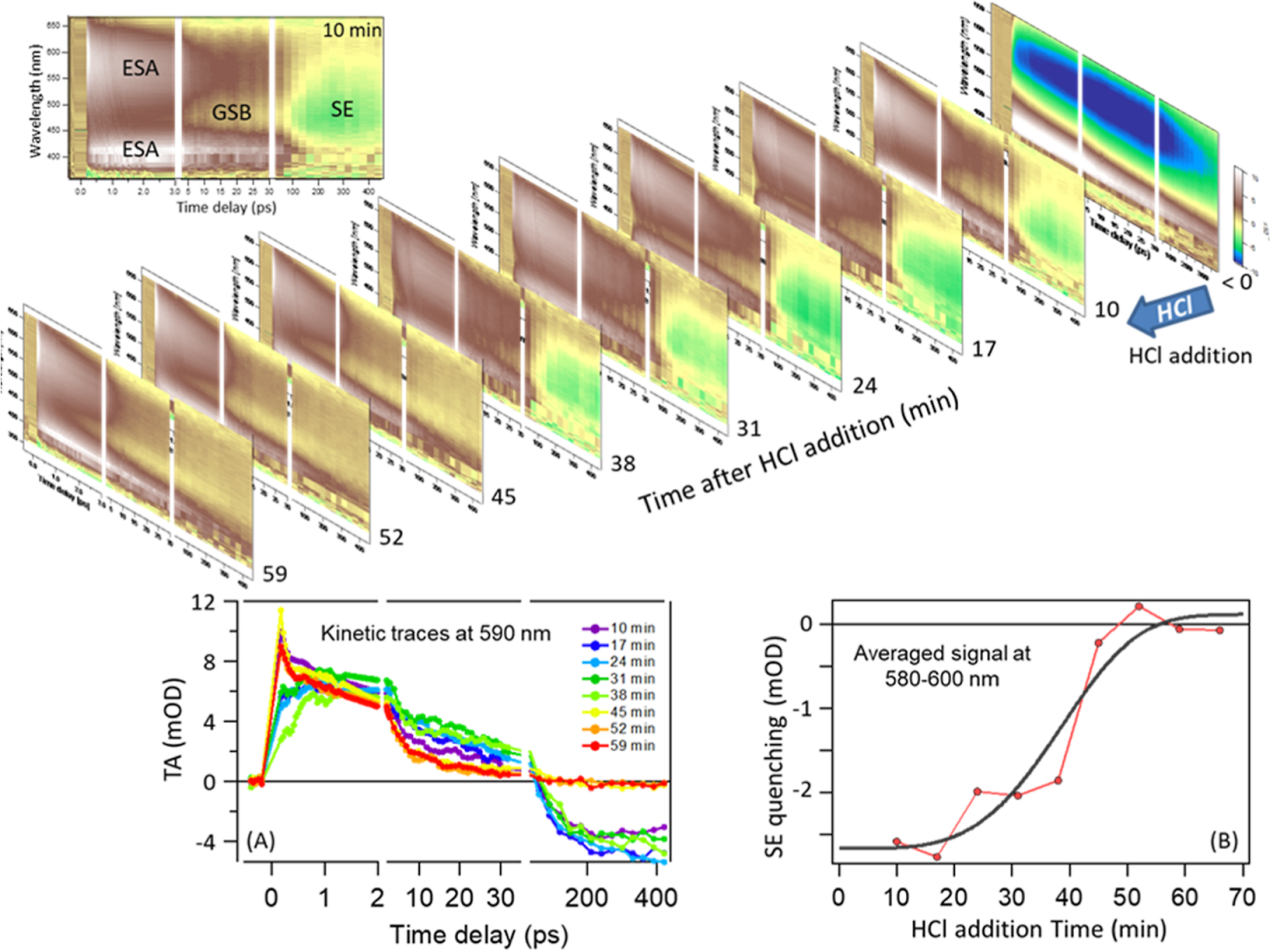
Main figure: a series of consecutive TA scans as a function of time after the addition of HCl. The first plot (<0) shows the signal of complex **1** (see ref 11). The strong negative (blue) signal corresponds to the ground-state bleach (GSB). For clarity, the signals of **1·HCl** (ESA, excited-state absorption, SE, stimulated emission) are marked in the first scan after HCl addition (10 min). Inset (A) Kinetic traces at 590 nm (where essentially SE only is monitored and there is no contribution from GSB) at subsequent times after HCl addition. Inset (B) SE quenching kinetics monitored as the amplitude of TA signal at 400 ps (the longest scanned delay time) averaging points between 580 and 600 nm, where the TA signal is mainly SE. The solid line is obtained by fitting the data to eq 1. Kinetic traces at other wavelengths are shown in Figure S9. The reported addition times correspond to the end of each scan, which lasts 7 min each and that after HCl addition, we waited 3 min for complete, homogeneous **1·HCl** formation. The sample was continuously exposed to 1 kHz 400 nm irradiation from minute 3 to 59.

Moving to the S-shaped kinetics, this is more characteristic of phase transformations upon nucleation and, assuming constant nucleation rate and temperature, a three-dimensional growth follows the Avrami equation:^13^

1 $$R_{V}=1-e^{-Kt_{ncl}^{4}}\left(t_{ncl}>0\right)$$

where *R*_V_ is the volume fraction of the final phase at nucleation time *t*_ncl_ and *K* is a constant proportional to the formation rate of nucleation centers and volume growth rate of the transformed material. As tacitly assumed in inset B of Figure 4, the SE amplitude, estimated as the TA amplitude at 590 nm, at the longest scanned time delay reports on *R*_v_ at a given *t*_irr_:

2 $$R_{V}=\frac{{TA}_{590nm}^{400ps}\left(t_{irr}\right)-{TA}_{590nm}^{400ps}\left(0\right)}{{TA}_{590nm}^{400ps}\left(\infty \right)-{TA}_{590nm}^{400ps}\left(0\right)}$$

where we consider the differential change because of the small long-lasting residual (although constituting <3% of the overall change). Data are indeed perfectly described by eq 1, corroborating the formation of a solid upon 3D heterogeneous nucleation as quenching mechanism (fitting coefficients in Table S3). As countercheck, we exposed a sample to steady-state 400 nm radiation, and indeed a dark green, nonemissive precipitate was formed (Figure S2B). To verify whether the aggregated molecules were photodamaged, we added NH_3_ to the mixture. Indeed, since NH_3_ can withdraw HCl from the adduct,^9^ and **1** is more soluble than **1·HCl**, the HCl-deprived precipitate can be redissolved. The resulting solution shows the same spectroscopic features as the freshly prepared sample, including the changes upon HCl addition. This definitively confirms that the quenching mechanism involves intact, unexcited **1·HCl** adducts and, to a lesser extent, molecules of **1** from de-excited adducts, discarding photodegradation and photofragmentation. With our fluence (3 × 10^13^ absorbed photons per second) and solute content (5 × 10^17^ molecules), the fraction of adducts that were excited, at least once, before the near-complete suppression of SE (*t*_irr_ = 40 min) is less than 15% (see the Supporting Information). Therefore, when the suppression of SE, which involves the majority of **1·HCl**, is observed, only about 15% of them have been excited. This implies that the quenching of SE is not caused by the depletion of the concentration of **1·HCl**. Instead, most of the aggregated molecules have never been excited and still bear HCl. Since the first step of nucleation is still diffusion limited, it should occur at μs times or longer, therefore involving de-excited molecules. The formation of aggregates of unexcited adducts is a fact, as observed in solutions stored in the dark for days. This indicates that **1·HCl** can aggregate thermally, although inefficiently, at room temperature. Unexpectedly, de-excited molecules accelerate aggregation from days to minutes. Since photoexcitation induces conformational changes, as observed in **1**,^11^ and significantly speeds up nanoparticle formation, we infer that the latter requires a specific configuration, achieved either thermally with low probability or efficiently through photoexcitation. The aggregate consists of nanoparticles because the circulated sample remained clear and with no scattering, allowing to exclude particles larger than hundreds of nm. Spectroscopic stability was also confirmed (Figure S10). These findings imply that de-excited molecules act as nucleation centers. Their configuration, different from the ones accessible from ***S***_**1**_ and the ***S***_**0**_, facilitates the initial cluster formation, thus representing heterogeneous nucleation, even though the seeds are chemically similar to the solute.

Quenching kinetics reveal that nK emission activation is inhibited in the solid. As further evidence, we found that the emission was undetectable at cryogenic temperatures, where the sample was a glassy matrix. These findings indicate a process requiring molecular mobility, such as photodetachment or photoinduced recombination, rather than solely intramolecular relaxations (in the latter case, we should even expect an increase of the emission at cryogenic temperatures). The 70 ps rise time of the emission is too fast for diffusional mechanisms, ruling out photoinduced recombination. We can also rule out any effect due to a variation of HCl concentration in solution because spectra were measured in an HCl excess (10 mM of HCl and 0.4 mM of solute); therefore, even if all the **1·HCl** would release HCl, the total HCl concentration change would be 4% at most.

The fact that the direct excitation of ***S***_**1**_ does not induce SE and (fast) aggregation, whereas the ***S***_**2**_ excitation does, unveils that the conformational change responsible of such different photochemical behavior is triggered during the short-lived non-Kasha state and continues to evolve after the IC to ***S***_**1**_. To identify the nature of the process induced by this photoisomerization, we observe that the emission must stem from ***S***_**1**_, which, however, would naturally emit at λ > 800 nm. The only process able to explain a so dramatic blue-shift is the opposite process which caused the decrease of the **S**_**0**_–**S**_**1**_ gap and the red-shifting of the steady-state absorption bands upon HCl addition: breaking of the adduct **1·HCl** with ejection of an HCl molecule. Indeed, as can be seen experimentally and computationally (Figure S1), the HCl release causes a blue-shift of almost 200 nm (from ca. 800 nm to 600 nm) of the lowest optical transitions. Ejection of Cl^–^ alone is excluded by TD-DFT calculations (see ref 9 and Figure S1C), in agreement with previous findings on similar tight ion-pair adducts which lose HCl instead of Cl^–^.^14^ Noteworthily, we observed a monoexponential SE rise with no evidence of intermediate processes (see Figure S14 and related discussion), supporting a dissociation process of the whole HCl molecule.

Thus, this study represents a rare case of non-Kasha photochemistry, rather than photophysics, where femtosecond processes initiate effects lasting hundreds of picoseconds to nanoseconds: in subpicosecond time scales: the ***S***_**2**_ excitation in **1·HCl** destabilizes the contact-pair site, leading to HCl detachment within tens of ps. The complex after the HCL release is chemically equivalent to **1** but differs from it, as it can trigger aggregation likely in μs and exhibit strong emission, both absent in **1**. It is worth mentioning that the formation of micro- and nanometric aggregates of HCl adducts of a platinum-bis-dithiooxamidate complex was earlier observed by Lanza, Campagna, and co-workers.^15^

Precipitate formation is the final step of a process beginning with the growth of nanosized particles, which stay in solution because of their small size and continuous flow. Therefore, the content of molecules before and after nucleation remains unchanged, explaining why the signal intensity in the first 10 ps is independent of nucleation (Figure 4). This proves that molecules in solid phase and solution are the same, and only after HCl ejection do the latter differ from the former. For these reasons, we previously stated that the aggregation needs photoisomerized molecules as seeds, but it contains nonexcited **1·HCl**.

We can therefore conclude that adducts **1·HCl** are not emissive, but the HCl photodetachment converts them into emissive species, with the “non-Kasha” emission originating from ***S***_**1**_. In this respect, it could also be described as nK photoinduced chemiluminescence, resulting from a chemical reaction happening or triggered in higher excited states. Accordingly, the emission quenching is caused by the depletion of free **1·HCl** by incorporation into aggregates, which are nonfluorescent because the HCl photodetachment is inhibited.

About the emissive behavior, direct excitation of ***S***_**1**_ of **1·HCl** shows no steady-state emission due to competitive ISC;^11,16^ therefore, since the HCl release makes the system emissive, it must also drastically reduce the ISC rates. This definitively points to a conformational change that would allow for strong emission. An extensive computational study is in progress to clarify the details of structural dynamics, but preliminary results reported in Section SI.8 (Figures S11 and S12 and related discussion) confirm that these systems have complex potential energy surfaces with different stable local minima,^11^ thermally and optically accessible. Importantly, we do not pretend to report a fully computational description of all the complex photophysical and photochemistry pathways, which would be out of the scope of the present contribution, but rather provide some argument to substantiate the previously sketched mechanism. Relevant here is that one of these minima, close to the equilibrium configuration and thermally connected to it, exhibits inversion of LUMO and LUMO + 1 with respect to the absolute minimum (Figure 3). This is plausible because in **1** these MOs are almost degenerate and even inverted in vacuum.^11^ This configuration is shown in Figure S12. The excited electron density of the charge transfer state ***S***_**1**_, after HCl ejection, would be exclusively localized on the quinoxdt centered LUMO moiety, without relevant Pt orbital contributions (Figure 3). This condition could increase the π-staking interaction of this ligand, explaining the initial aggregation. Furthermore, the suppression of Pt contribution, negligible overlap between the orbitals occupied by the excited electron and the hole, and the same π symmetry of these two singly occupied orbitals (which would make ISC forbidden) could drastically reduce the spin–orbit coupling, thereby accounting for the emissive behavior of the photoisomerized complex. This is supported by the preliminary calculations reported in Section SI.8 of the Supporting Information (Tables S7–S9, and relative discussion), where we also note that the ***S***_**2**_ is quasi degenerated with several higher triplet states, with the closest triplet state being at ca. 90 cm^–1^. Remarkably, upon 400 nm excitation, the sample after 59 min of irradiation produces TA signals similar to the 800 nm excitation (Figure S8), except for a small long-lived negative signal at 500–600 nm. This means that when HCl cannot be released, adducts follow the proposed nK emission mechanism of **1**. Concerning the long-lived signal, its presence is confirmed by nanosecond time-resolved measurements on photoaggregated **1·HCl** (Figure S2A). This suggests that the long-lived signals arise from a small fraction of **1·HCl** in the aggregate that can still release HCl, as for instance on the surface or in voids (or less dense regions) of the aggregates or a small fraction of **1·HCl** dissociated from the aggregates. Very likely, these adducts can undergo geminate (in the voids) or nongeminate (on the surface or in solution) recombination, giving a constant signal over time.

These preliminary computational results also help identify possible conformational changes that could transform photoexcited molecules into nucleation centers and promote the stacking of unexcited molecules. In particular, the local minimum configuration that is thermally accessible from the global minimum (Figure S12 and related discussion) is the most plausible candidate for the isomer responsible for precipitate formation. In this conformation, the two benzyl rings in the dithiooxamidate moiety are oriented more parallelly and ordered, which could facilitate the formation of packed and ordered aggregates and ultimately of nanoparticles.

Before concluding this section, we should comment on the involvement of triplet states in the nK emission. Due to heavy atom effects, on the tens-of-ps to ns time scale, triplet states are very likely populated,^11^ as also supported by the calculated spin–orbit coupling (Tables S7–S9, and relative discussion). This could allow for an indirect mechanism of delayed fluorescence, where the emissive state is repopulated from the triplet (dark) states. However, as detailed in the Supporting Information (SI.10), even if we cannot exclude such a process, it would not alter the main conclusion that our experimental results speak for an allowed transition with a long lifetime.

## Conclusions

In this study, the intriguing photochemical properties of the **1·HCl** adduct are presented, complementing our previous work without HCl. We demonstrated that the excitation of long-lived (∼1 ps) higher excited states triggers conformational changes. They continue to evolve after the IC to ***S***_**1**_ and to long-lived triplet states, as already observed in **1**,^11^ and can trigger high-yield chemical reactions. Therefore, the system investigated herein is the archetype of a new promising family of efficient multiresponsive materials, leveraging non-Kasha photoisomerization for excitation-dependent photochemical responses. Different mechanics of different nature concur to define the photochemical properties of the system: (1) a non-Kasha photoinduced chemiluminescence and (2) molecular aggregation upon nucleation with the photoexcited molecules themselves acting as nucleation centers.

The “non-Kasha” chemiluminescence is caused by the HCl detachment on ∼70 ps, triggered by subps conformational changes occurring in the higher excited states. This veritable non-Kasha chemical reaction triggers a dramatic ***S***_**1**_–***S***_**0**_ blue-shift and brings the molecule toward a configuration, which is emissive and responsible of turning photoexcited molecules into a nucleation center for unexcited molecules. This causes initially the formation of nanoparticles and eventually a precipitate. In this phase, molecules cannot release HCl and remain nonemissive (QY ∼10^–3^), in contrast to non-Kasha photoinduced chemiluminescence (QY > 10^–1^).

This result is groundbreaking for several reasons: (1) it is the first evidence of nonintramolecular photochemical reaction selectively induced by non-Kasha excitation with high yield, (2) it demonstrates that fs and subps dynamics can drive processes on much longer time scales not only in biomolecules, and (3) this way to exploit a non-Kasha response represents a radical change of view, paving the way to the emerging field of a veritable non-Kasha photochemistry.^2^ Unlike the aforementioned prevailing approaches, the functional photoprocess (HCl release) occurs on the long-lived first excited state, modified by non-Kasha photoisomerization. We introduced the term “beyond-Kasha photochemistry” to emphasize this special situation where we can switch between “Kasha” reactions (namely, occurring on the lowest excited state of a given multiplicity) by non-Kasha changes.

These results open new aspects to investigate, as identifying the nK conformers, the relevant features of the singlet and of triplet potentials energy surfaces and the formation and growing mechanisms of the molecular solid. It would also be worth investigating the capability of the solvent to modulate the nK activity, and the presence of specific low-frequency modes, vibrationally coupled to modes enabling HCl dissociation and further conformational changes, which could be populated upon relaxation toward ***S***_**1**_. Before concluding, it is worthy of notice that although the experimental evidence and the computational data reported here allow us to derive our interpretations, they do not allow us to draw conclusions about the specific deactivation paths and therefore about some of the fundamental mechanics underlying the observed processes. Indeed, in metal complexes, the relaxation of high-energy excited states can involve ultrafast ISC and even recrossing between states of different spin multiplicities, processes that might populate long-lived, high-energy singlet or triplet states. In this respect, the rather strong values of spin–orbit couplings reported in the SI speak to an important role of the triplet states in defining the deactivation paths. For such a thoughtful investigation, a nonadiabatic computational approach is mandatory to identify the possible conformers, the relaxation paths from the different excited states, and the role of external parameters, as the solvent and the specific acid. This extensive computational study, which is out of the scope of this review, is ongoing.

## Materials and Methods

### Sample Preparation

The complex *n*-Bu_4_N­[Pt­((*R*)-α-MBAdto)­(quinoxdt)] (*n*-Bu_4_N­[**1**]) was synthesized and characterized as described in ref 9. The solvent used for optical measurements was acetonitrile of spectroscopic quality. 2 mL of solution was prepared with concentration *c* = 4.4 × 10^–4^ M, corresponding to an optical density of 0.1 OD at 800 nm and 0.04OD at 400 nm in 200 μm. To form the **1·HCl** adduct, a solution of 10 mM of HCl in acetonitrile was added to the solution of complex **1**, until a [**1**]/[HCl] ratio of 1:3 was reached. The effect of HCl addition on the optical spectra is discussed in ref 9 and summarized for convenience in the Supporting Information.

### Optical Characterization

The UV–vis–NIR absorption spectra of **1·HCl** in acetonitrile solution were acquired with an Agilent Cary 5000 spectrophotometer using a 10 mm path length quartz cuvette. Emission and excitation spectra were collected with an Edinburgh Instruments FLS1000 photoluminescence setup equipped with extended PMT980 and nitrogen-cooled PMT1700 photomultiplier tubes. A 450 W CW xenon lamp was used for steady-state spectra. Time-Correlated Single Photon Counting (TCSPC) measurements were performed by using a pulsed 375 nm EPL laser (average power 150 μW, repetition rate 20 MHz, and pulse width 75 ps) on a freshly prepared solution in acetonitrile. Data were acquired after 10 min of accumulation. After the measurement, the incipient formation of a precipitate was noted.

### TA Measurement

The setup is described in detail in the Supporting Information and ref 17. Shortly, it is an ultrafast transient absorption (TA) setup with single-shot referenced detection operated with a 1 kHz femtosecond pulsed laser source (fundamental at 800 nm, 10 nm bandwidth, and 100 fs pulse length). Samples were excited at 800 nm (100 nJ/pulse into 60 μm diameter spots), close to the maximum of the lowest absorption band, and at 400 nm (65 nJ/pulse into 60 μm diameter spots), close to the maximum of the excitation band responsible of the 550 nm non-Kasha emission (Figure 1). With this setup configuration, a whole time-wavelength TA 2D plot (TA spectra at different time delays) with an acceptable signal-to-noise ratio was obtained in ca. 7 min. A power dependence measurement of the pump was regularly carried out before data acquisition to ensure that experiments were conducted in a linear absorption regime. After correction for probe group velocity dispersion, data from −180 fs to +180 fs around time zero were neglected to avoid artifacts caused by pump–probe cross-phase modulation from the solvent (see the Supporting Information).

Solutions were circulated in a closed flow circuit through a UV-grade flow-cell with a 200 μm-thick channel. The flow speed was set to 60 μL/s, which allows operating in a single-shot per spot regime to prevent signal artifacts and sample photodegradation due to multiple excitations and photoaccumulation. The TA experiments were always carried out on freshly prepared samples, with and without HCl addition (in the text, **1·HCl** and **1**, respectively).

In an aprotic solvent, such as acetonitrile, the HCl molecule is not expected to be dissociated. Moreover, these adducts can lose HCl instead of Cl^–^ since the hydrogen atom is more strongly linked to the chloride ion in comparison with the amidic nitrogen one.^14^ This implies that very likely the adduct formation is not a two-step process but involves an interaction between the complex and a whole, undissociated HCl molecule. More relevant for this study, this also guaranties us that the sample is not a mixture of only protonated **1** and **1·HCl** but it is only made of the latter.

To point out and monitor photoaccumulated effects, a small volume (2 mL) of **1** solution was placed in the setup, and HCl was added in situ. To have a uniform addition, the sample was circulated in the dark for 2 min. After the check for excitation linearity (∼1 min), 8 consecutive sets of experiments were acquired upon 400 nm excitation.

## Supplementary Material



## Notes

Transient absorption setup; effects of HCl addition; photocycle upon 800 nm excitation; comparison of the earlier transient absorption signal upon 800 and 400 nm excitations; comparison of the TA signals of the fresh sample upon 800 nm excitation and of a photoexposed sample upon 400 nm excitation; fitting of the quenching kinetics with the Avrami equation; calculation of the fraction of excited molecules; preliminary results from calculations; global fit analysis of the traces from panel A of Figure 4; and comment on the involvement of triplet states in the non-Kasha emission (PDF)

## ¶

College of Science and Engineering Hamad Bin Khalifa University, Education City, Doha 34110, Qatar (S.S.A.)

## ∇

Dipartimento di Ingegneria Civile, Ambientale e Architettura, University of Cagliari, I-09123 Cagliari, Italy (P.D.).
