# Supplementary material for: Beyond-Kasha Photochemistry in a Heteroleptic Platinum–Dithiolene Complex
Source: J Am Chem Soc. 2026 Mar 13;148(11):12186–93. doi: 10.1021/jacs.6c00565 (PMC13022878; doi:10.1021/jacs.6c00565)
Supplement: Supplementary file 1 [file ja6c00565_si_001.pdf]

Supplementary information

Beyond-Kasha Photochemistry in a heteroleptic platinum-dithiolene complex

Michela Gazzetto,[1] Flavia Artizzu,[2] Salahuddin. S. Attar,[3] Jakob T. Casanova,[1] Luciano Marchiò,[4] Luca Pilia,[5] Antonio Monari,[6] Paola Deplano,[3*] Andrea Cannizzo[1*]

[1] Institute of Applied Physics, University of Bern, CH-3012 Bern, Switzerland. [2] Department of Sustainable Development and Ecological Transition (DISSTE), University of Eastern Piedmont, I-13100 Vercelli, Italy. [3] Dipartimento di Scienze Chimiche e Geologiche, Università di Cagliari, I-09042 Monserrato (CA), Italy. [4] Dipartimento di Scienze Chimiche, della Vita e della Sostenibilità Ambientale, Università di Parma, I-43124 Parma, Italy. [5] Dipartimento di Ingegneria Meccanica, Chimica e dei Materiali, Università di Cagliari, I-09123 Cagliari, Italy. [6] Université Paris Cité and CNRS, ITODYS, F-75006, Paris, France. [7]

*corresponding authors: andrea.cannizzo@unibe.ch, deplano@unica.it.

SI.1 Transient Absorption set-up 20

SI.2 Steady state and ns time resolved optical measurements and effects of HCl addition 22

SI.3 Investigation of the photocycle upon 800 nm excitation 26

SI.4 Comparison of the earlier transient absorption signal upon 800 nm and 400 nm excitations 28

SI.5 Comparison of the TA signals of the fresh sample upon 800 nm excitation and of a photo-exposed sample upon 400 nm excitation 31

SI.6 Fitting of the quenching kinetics with the Avrami equation. 32

SI.7 Calculation of the fraction of excited molecules 33

SI.8 Preliminary Results from calculations 34

SI.9 Global fit analysis of the traces from panel A of Figure 4 43

SI.10 Comment on the involvement of triplet states in the non-Kasha emission 45

References 46

SI.1 Transient Absorption set-up

The 800 nm pump pulse was generated by 1 KHz Ti:Sapphire regenerative amplifier (Coherent Elite Duo Femto). Energy per pulse was 100 nJ at the sample position and was focused into a ca. 60 µm diameter spot size ($1/e^2$). The 400 nm pump pulse was obtained frequency doubling with a thin 0,25 mm BBO the 800 nm pump pulse. Energy per pulse was 65 nJ at the sample position with a focus of c.ca 60 µm. The probe pulse was a broadband continuum, generated by focusing a fraction of the Ti:Sapphire fundamental into a 5 mm thick CaF_2 crystal. The relative delay between pump and probe was delayed by a computer-controlled delay stage (Nanotec Electronic with PS 35 controller stage by OWIS GmbH) in the pump beamline. A referenced detection scheme was adopted: the continuum pulse was split by a 50/50 beam splitter into two beams, which were then focused into two different spots of the sample with ca. 40 µm diameter spot size. Only one of the two spots was overlapped with the pump pulse, while the other was kept unperturbed for referencing (in the following probe and reference, respectively). After the sample both probe and reference are collimated by an achromatic doublet lens, dispersed by a grating and then focused into two fast CMOS cameras capable of measuring individual shots (Glaz PulseSync, Synertronic Designs). Spectral- and amplitude fluctuations of the continuum were then corrected shot-per-shot by dividing the probe spectrum by the reference one. A reference baseline for pump-reference ratio was recorded for each measurement by introducing a mechanical chopper in the pump beamline at 0.5 kHz to block every alternate pump pulse. A photo-diode after the chopper was used to monitor shot to shot pulse intensity in order to sort out 'pumped' and 'unpumped' measurements and to compensate for fluctuations and drifts of the pump pulse intensity. All these corrections were applied to single-shot TA signals ($TA_i(t,\lambda)$) before averaging over 2000 shots for each pump-probe time delay:

$$TA(t,\lambda) = \frac{1}{ln(10)}\left[1 - \frac{p_0}{p}\left(\frac{I_{s,1}(t,\lambda)}{I_{r,1}(t,\lambda)}\right) \div \left(\frac{I_{s,0}(t,\lambda)}{I_{r,0}(t,\lambda)}\right)\right]$$

with $I(t,\lambda)$ the spectrum recorded by the camera averaged on 2000 shots. The index *s* and *r* refer to the spectra recorded by the camera monitoring the probe and reference pulses, respectively. The index 1 and 0 refer to whether the indexed spectrum was pumped or unpumped respectively. The sorting criterion as stated before, is provided by a photo-diode. The normalization is accounted for by p_i which is the pump intensity recorded by the second photo-diode that is also indexed in the same fashion; since p_0 is a reference value of pump intensity collected at the very beginning of the scan the fraction p_0/p_i takes into account both the shot-to-shot fluctuations and the slow drifts of the laser intensity.

The signal was then calculated accordingly to the following equation,

$$TA(t,\lambda) = \frac{1}{n}\sum_{i=1}^{n} TA_i(t,\lambda)$$

A power dependence measurement was regularly carried out to ensure that experiments are conducted in the linear absorption regime.

SI.2 Steady state and ns time resolved optical measurements and effects of HCl addition

Upon adding HCl, the color of the solution of [Pt((*R*)-α-MBAdto)(quinoxdt)]$^-$ complex in acetonitrile changes from deep blue to green (Figure S1A). A substantial change in the absorption is observed as shown in Figure S1A. The lowest absorption band at 600 nm disappears, while a new one is formed around 800 nm. The process varies with addition of HCl aliquots and it is complete for a 1:1 molar ratio between HCl and the complex. As reported in the main text, this is due to the formation of a tight adduct between HCl and the dithioxamidato ligand (MBAdto), where N··H··Cl interaction stabilizes the ion-pair. The formation of tight-contact ion-pairs between N,N′-dialkyldithiooxamidate Pt-Pd metal complexes with HCl has been extensively investigated by Lanza, Campagna and coworkers.[18] Further support on the nature of these tight-contact pairs has been provided by a structural characterization of a related Rh complex, ([[(C_5Me_5)ClRh(H_2-isopropyl$_2$DTO k-S,S Rh)]$^+$Cl$^-$)·$CHCl_3$,[19] showing that the two amidic N-H moieties of the coordinated dithiooxamide act as hydrogen bond donor groups towards a chloride ion. The obtained values of bond lengths, bond angles and directionality of the ionic hydrogen-bonding (IHB) compared with literature data[20 21] can explain the formation of associated tight ion-pair that only polar solvents with donor atoms such as Me_2SO, MeOH are able to remove HCl from tight ion-pair.[18, 19-21] Our results are fully consistent with these findings and are supported by computational studies. The calculated absorption spectrum, indeed, nicely matches the experimental one (Figure S1 and Table S1). The emission colour changes from 720 nm to 570 nm with a higher emission quantum yield (Φ) of 1.4x10^{-4}. The excitation spectrum at $\lambda_{emission}$=570 nm is peaked at around 410 nm after HCl addition. Also in this case no photoluminescence is observed upon excitation of the lowest absorption band, pointing to a non-Kasha emission behavior.

Time-resolved emission measurements in acetonitrile solution in the presence of HCl reveal a biexponential decay dynamics (Figure S2A and Table S2) in the ns range.

The sequence of frontier molecular orbitals (MOs) in the presence of HCl (Figure S1C) does not change and it is still confirmed the charge-transfer character of the transitions. Indeed, the HOMO → LUMO transition (HOMO = highest occupied molecular orbital; LUMO = lowest unoccupied molecular orbital), which corresponds to the ground state → S_1, is still a ligand-to-ligand charge transfer to MBAdto moiety. This transition is stabilized from the presence of HCl and peaked at around 800 nm, with the central Pt more involved in the molecular orbitals respect to the case of **1**(Figure S1A). The ground state → S_2 transition is namely a HOMO-1 → LUMO with a very small oscillator strength. The next strongly allowed transition is the ground state → S_3, which is a HOMO → LUMO+1 transition with a (mixed metal) intra-ligand charge transfer character to the quinoxdt moiety.

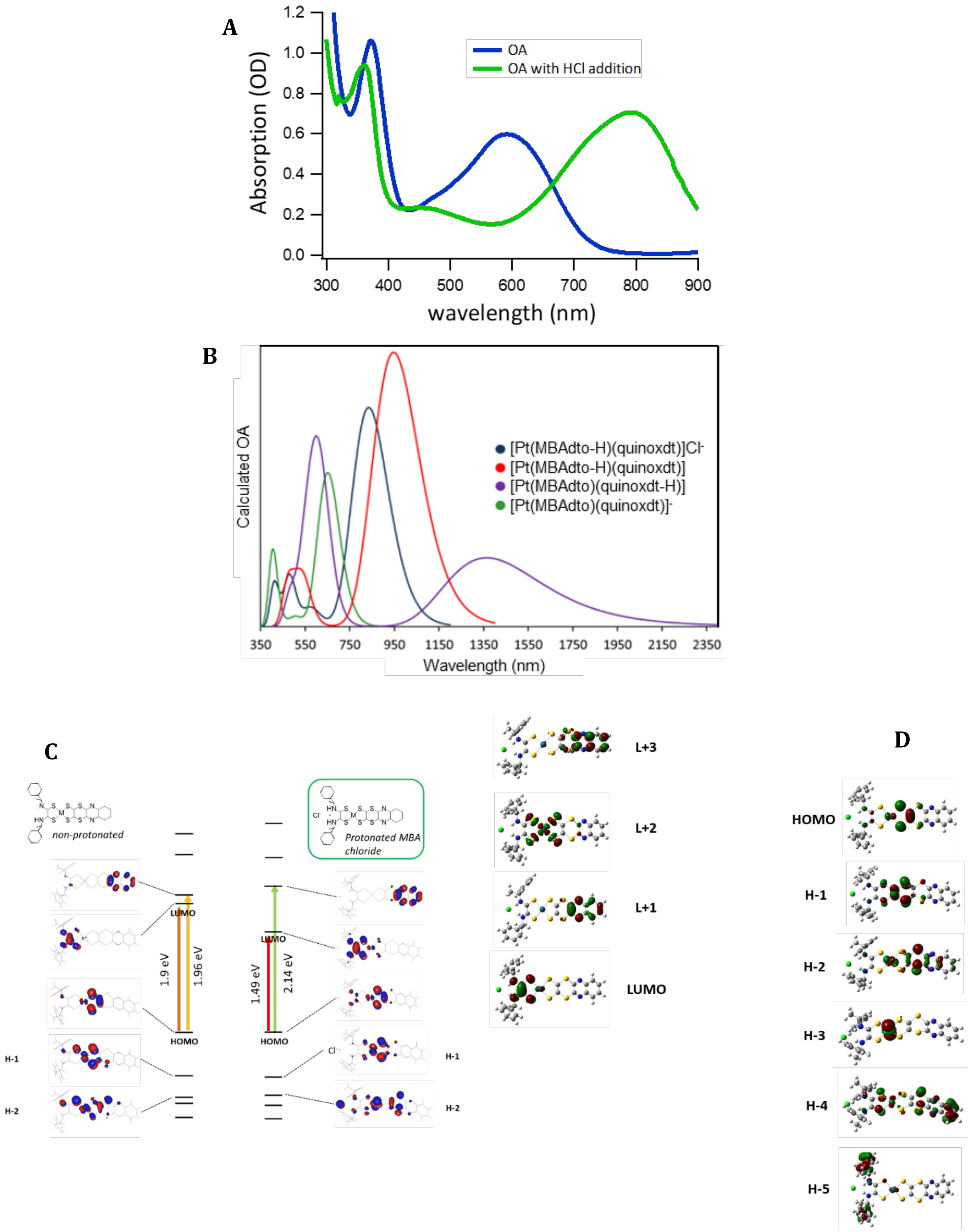

Figure S1: A) UV-Vis optical absorption (OA) spectra of $^{-}$complex **1** with and without HCl addition. B) Simulated UV-vis spectra for the first ten singlet to singlet transitions of [Pt(MBAdto-H)(quinoxdt)]Cl^{-} or **1**·**HCl** (blue line), [Pt(MBAdto-H)(quinoxdt)] (red line), [Pt(MBAdto)(quinoxdt-H)] (purple line), [Pt(MBAdto)(quinoxdt)]$^{-}$ (green line). C) Comparison of energies of the calculated molecular orbitals (MOs) of 1 and **1**·**HCl** in solution. D) DFT calculated MOs of **1**·**HCl** $^{-}$ in acetonitrile. Molecular orbitals and structures adapted from ref. 9, copyright 2017 American Chemical Society.

Other strongly allowed higher excited state transitions are HOMO-2 → LUMO and HOMO-5 → LUMO, which are centered in the spectral region where is found the excitation band of the non-Kasha emission is found. The first one is a MMLCT, with transfer of charge density from mixed Pt-quinoxdt molecular orbital to the MBA moiety, while the second one is an intra-ligand transition localized on the MBAdto moiety, where the tight contact-pair is located.

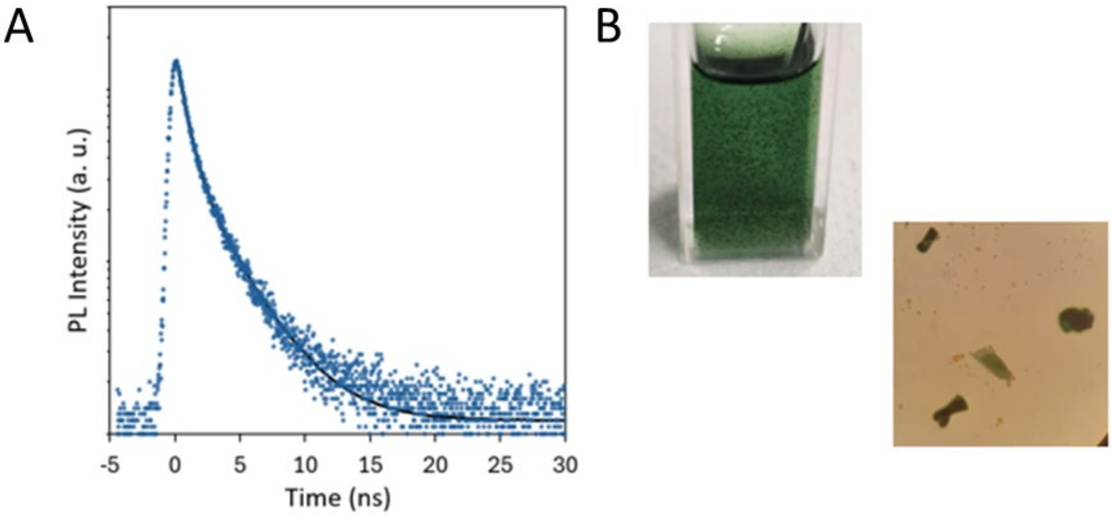

Figure S2: a) Time-resolved emission decay curve of **1·HCl** (blue dots). The solid black line represents the best fit to data for a biexponential decay (Table S2). λ_{exc} = 375 nm, λ_{em} = 560 nm; b) Photographs of the aggregate particles formed after irradiation. The optical microscope image shows that the particles are constituted by green crystals.

To investigate the non-Kasha behaviour of the complex we carried on fs TA experiment in HCl solution with ratio 1:3 [**1**]:[HCl]. At first we have excited the lowest transition to S_1 upon 800 nm excitation, than we performed the experiments upon 400 nm excitation, corresponding to excitation of the non-Kasha emission.

Table S1: TD-DFT calculated energies and compositions of the lowest lying singlet electronic transitions **1·HCl** - in the solution phase, acetonitrile, (B3LYP/6-31+G(d)-SDD). The principal singlet transitions responsible for the main absorption band in the visible-NIR region are shown in bold, while the ones mainly responsible of the non-Kasha emission are underlined.

State	Composition[a]	ΔE(eV/nm)[b]	f [c]	Character
**1**	**HOMO → LUMO, 100%**	**1.49 / 835**	**0.2304**	quinoxdt /Pt → MBAdto/Pt
2	HOMO-1 → LUMO, 98%	1.97 / 630	0.0008	
**3**	**HOMO → LUMO+1, 99%**	**2.14 / 578**	**0.0175**	quinoxdt /Pt → quinoxdt
4	HOMO → LUMO+2, 97%	2.20 / 558	0.0019	
5	HOMO-3 → LUMO, 89%	2.27 / 547	0.0005	
<u>6</u>	<u>HOMO-2 → LUMO, 88%</u>	<u>2.58 / 481</u>	<u>0.0541</u>	quinoxdt /Pt → MBAdto /Pt
7	HOMO-1→ LUMO+2, 94%	2.83 / 438	0.0003	
8	HOMO-4 → LUMO, 66%<br>HOMO-12 → LUMO, 20%	2.91 / 426	0.0024	
<u>9</u>	<u>HOMO-5 → LUMO, 83%</u>	<u>3.01 / 412</u>	<u>0.0454</u>	MBAdto → MBAdto/Pt

Table S2. Time-resolved PL data for **1·HCl**.

τ_n (ns)	Contribution (%) [a]	τ_{av} (ns)[a]
0.777(8)	39	2.20
3.13(5)	61	

[a] Intensity average lifetime calculated as $\tau_{av} = \frac{\sum a_i \tau_i^2}{\sum a_i \tau_i}$

SI.3 Investigation of the photocycle upon 800 nm excitation

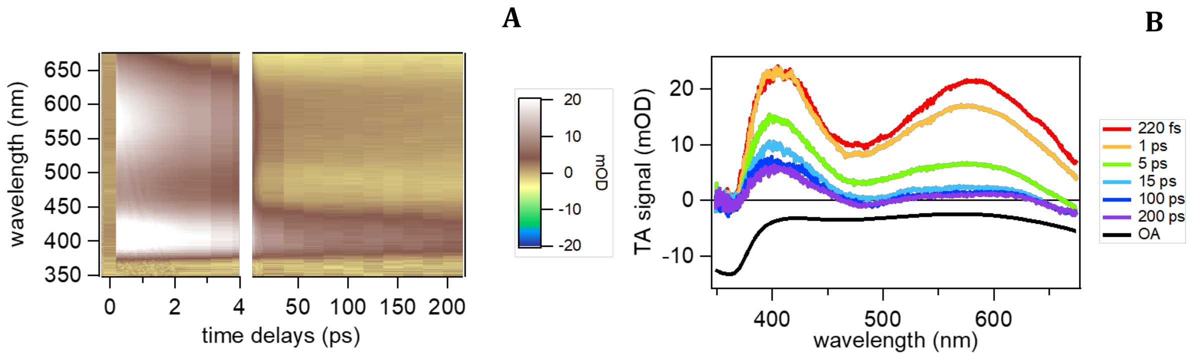

Figure S3: A) fs TA time-wavelength plot of **1·HCl** upon 800 nm excitation in acetonitrile. B) Spectra at selected time delays from panel A). The steady-state absorption spectra are reported inverted to match the TA spectra and to help the assignment of the transitions.

Figure S3A reports the fs TA experiment upon 800 nm excitation and Figure S3B shows the spectra at selected time delays. Two strong ESA bands develop within the instrumental time resolution, peaked at 410 nm and 580 nm, respectively. The negative bands centered at 360 nm, 480 nm and in the low energy region (λ_{probe} > 650 nm) closely match the inverted absorption spectrum and are therefore attributed to GSB. It has to be noted that in this situation we cannot distinguish if the ESA bands are effectively one or more. It could be that we are observing one sole broad ESA band peaked at 480 nm, overlapping with GSB also peaked at 480 nm, as well as we are observing two or more ESA bands. Before any analysis we can observe that the excited state lifetime must be in the ns range, confirming what was expected in literature from steady state analysis.

By means of SVD data analysis, TA were spectrally decomposed into four DAS, shown in Figure S4, each decaying with its own lifetime: 0.9 ps, 3.9 ps, 120 ps and ∞. DAS τ_1= 0.9 ps describes an ESA energy redistribution, with a decay of the ESA peak at 580 nm and a growth of the one at 410 nm. If we make the hypothesis that we are observing just one ESA band, DAS τ_1 describes a blue shift of the ESA. Considering the timescale, this mechanism can be safely attributed in both cases to vibrational cooling in the S_1 state. DAS τ_2= 3.9 ps describes an overall decay of the whole signal, with a stronger decay of the ESA at lower energies. Considering the fact that the complex is not emissive and confronting the result with our previous analysis for just the heteroleptic complex, we assign these dynamics to ISC and cooling in the triplet excited state. DAS τ_3= 120 ps describes a decay of the overall signal without any change in the spectral pattern. Both the 100s of ps time scale and the spectral evolution is typical of rotational diffusion. Accordingly, we can safely assign this DAS to such a process. DAS τ_4 is modelled with a step function and describes ground state

recovery with a contribution to the dynamics much longer than the scanned interval, pointing to excited state lifetime in the ns range as previously observed.

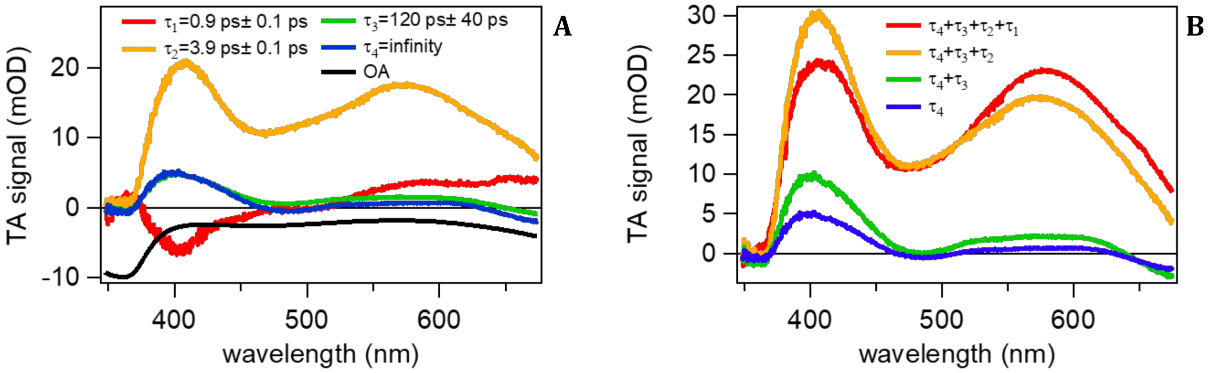

Figure S4: A) DAS obtained by SVD analysis of fs TA experiment upon 800 nm excitation. Each curve is labelled with the respective time constant. τ_4 describes a contribution with dynamics much longer than the scanned interval and is modelled with a step function. B) DAS sums reconstructing the experimental dynamics.

This is substantially identical to what found with complex **1** upon excitation within the lowest absorption (615 nm)[11]. The only difference is the spectral shape between 500 nm and 650 nm and can be explained as a shift of the GSB from 610 nm to 680 nm. The study on the **1·HCl** at 800 nm shows that the presence of the HCl has no effect on the excited state and its dynamics.

SI.4 Comparison of the earlier transient absorption signal upon 800 nm and 400 nm excitations

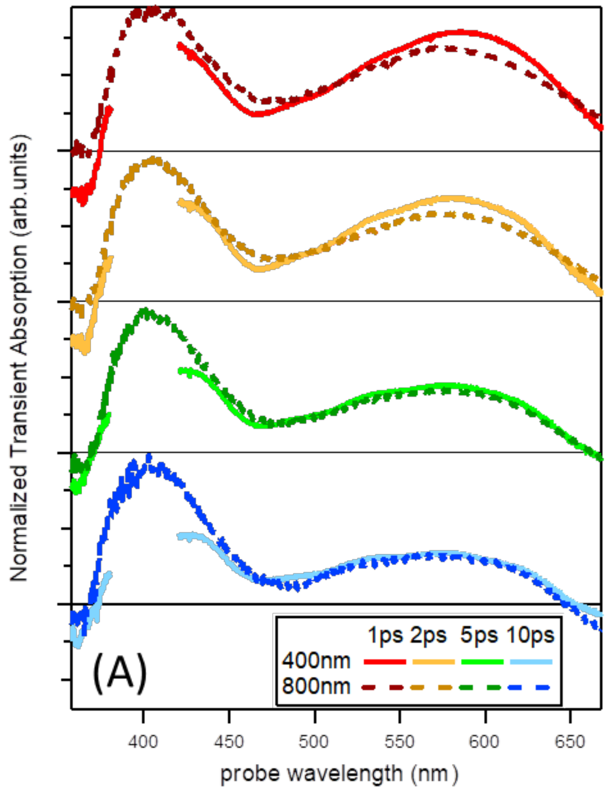

Figure S5. Comparison of TA spectra representative of the spectral evolution in the range 1 to 10 ps, namely after all the internal conversion processes and before of the delayed emission. For sake of comparison spectra are vertically shifted and normalized to match in the region 500-650 nm. Horizontal black lines are the respective zero lines.

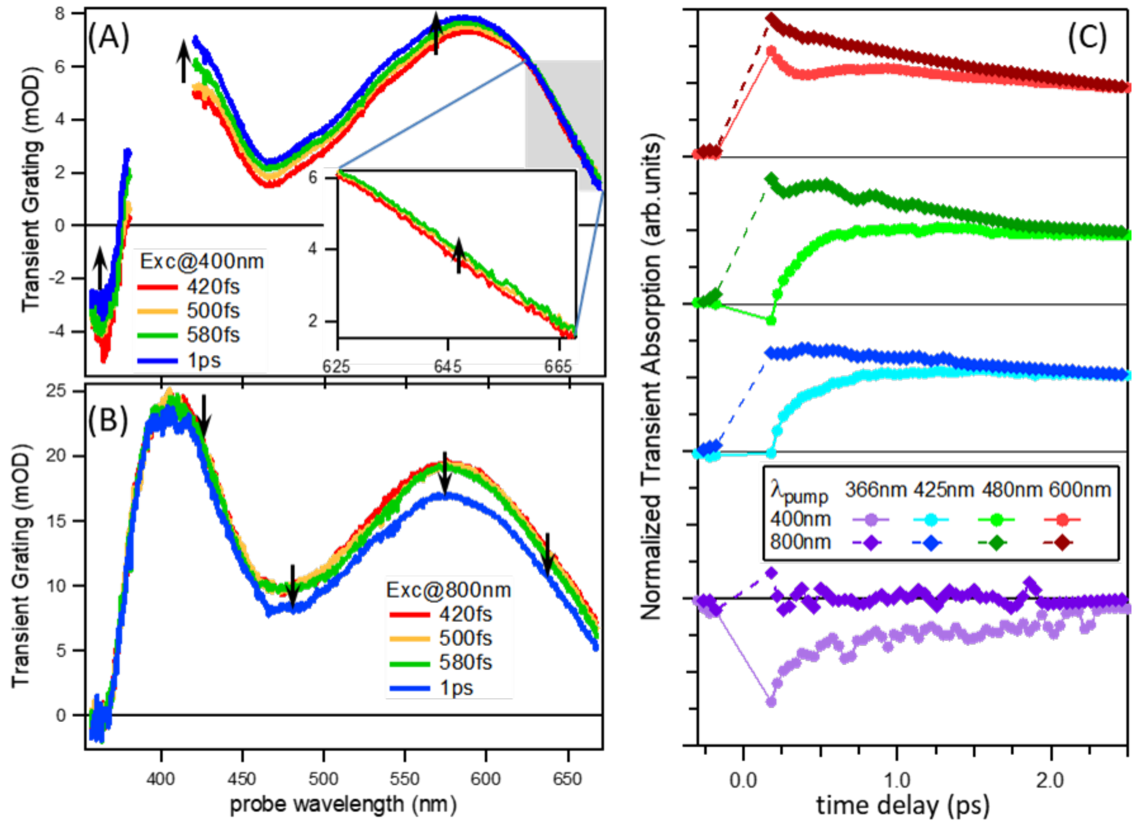

Figure S6. Comparison of representative TA spectra upon excitation at 400 nm (A) and 800 nm (B) to show the sub-ps rise (decay) of the signal upon 400 nm (800 nm) excitation, as highlighted by the black arrows. Inset in panel (A) zooms in the range at λ>625 nm to show that the rise upon 400 nm excitation is ubiquitous. The presence of the rise only upon 400 nm excitation is clearly observed by comparing kinetic traces at selected wavelengths. For the sake of comparison, kinetic traces are normalized to overlap in the ps timescale.

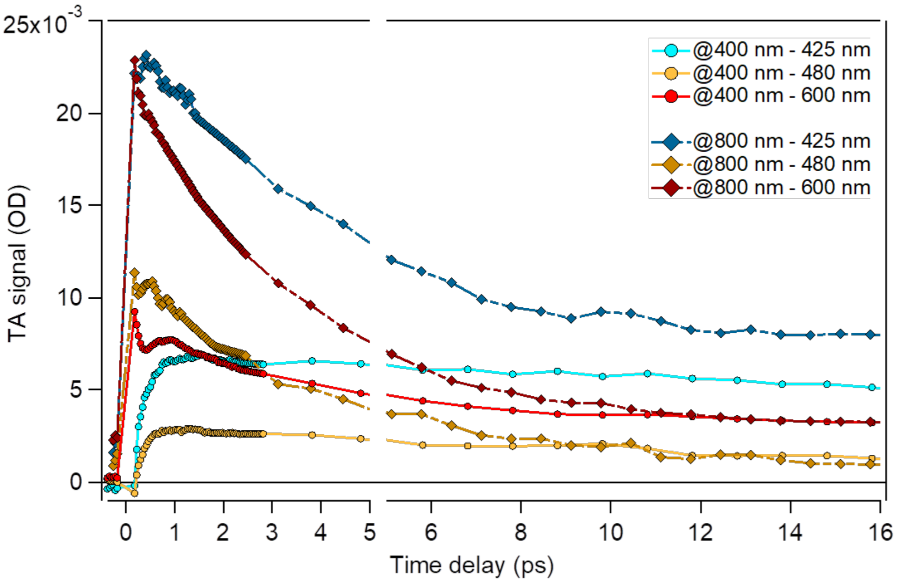

Figure S7. Comparison of representative TA kinetic traces upon excitation at 400 nm and 800 nm on a fresh sample.

SI.5 Comparison of the TA signals of the fresh sample upon 800 nm excitation and of a photo-exposed sample upon 400 nm excitation

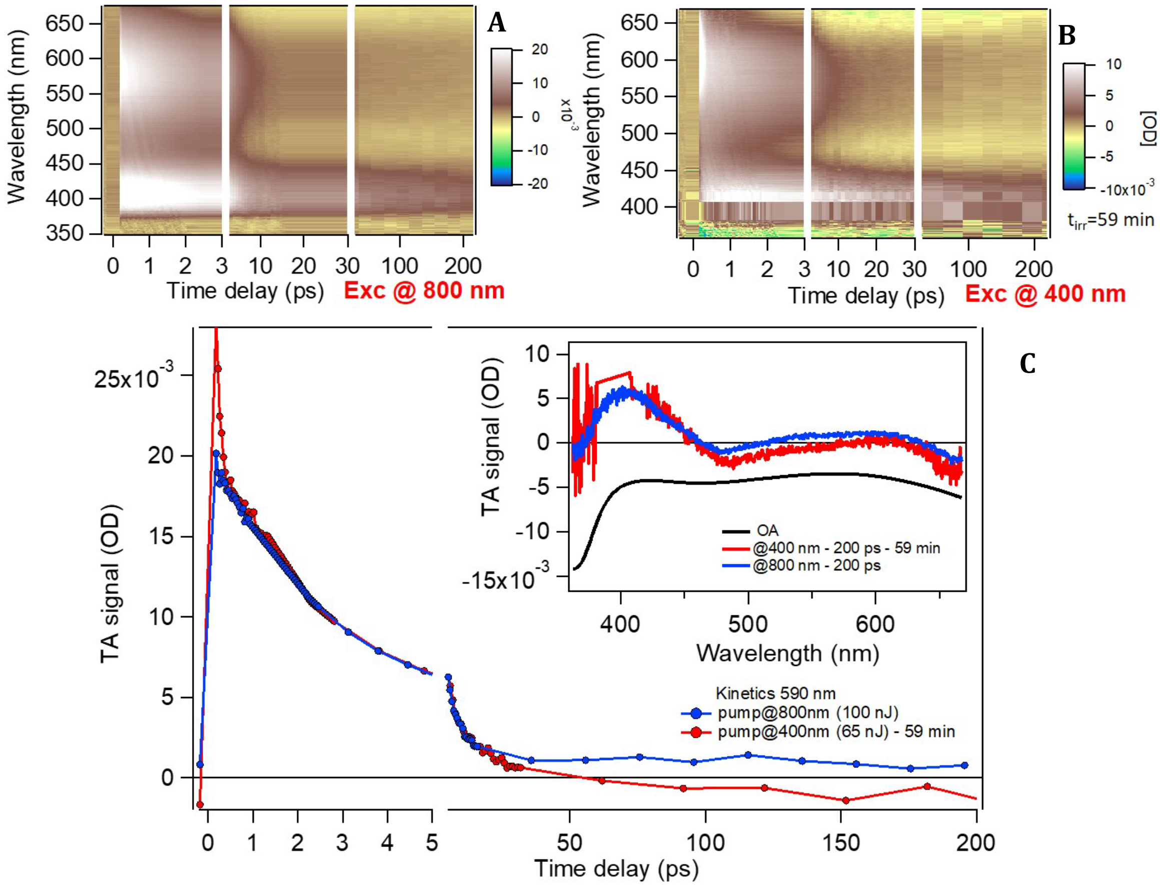

Figure S8. 2D TA signals (A) upon 800 nm excitation on a fresh sample and (B) upon 400 nm on a sample after 59min of irradiation, which corresponds to a completed SE quenching dynamics (see Figure 4 and Figure S9). (C) Kinetic traces at 590 nm and (inset) the spectra at 200 ps extracted from the two 2D plots. The two data sets are essentially the same except in the earlies, sub-ps, time range and at the latest times, where the irradiated sample still shows a very small negative signal, revealing a very small emission signal.

SI.6 Fitting of the quenching kinetics with the Avrami equation.

According to the Avrami equation (eq. 1) and eq. 2, we fitted data in in panel B of Figure 4 and Figure S9 with the function:

$$TA_{SE}(t_{irr}) - TA_{SE}(\infty) = \left(TA_{SE}(\infty) - TA_{SE}(0)\right) \cdot \left[1 - e^{-Kt^4}\right] = -\Delta \cdot e^{-Kt^4} + TA_{SE}(\infty) \quad \text{eq. S1}$$

where we replaced t_{ncl} with t_{irr} and, for sake of readability, TA_{590nm}^{220ps} with TA_{SE}. The fitting coefficients are reported in Table S3.

λ_{Pm} (nm)	550	580	605	$\langle 580-600 \rangle$ (from Figure 4B)
Δ (mOD)	$(4.9 \pm 0.5)\ 10^{-3}$	$(3.2 \pm 0.5)\ 10^{-3}$	$(2.5 \pm 0.5)\ 10^{-3}$	$(2.8 \pm 0.3)\ 10^{-3}$
$TA_{SE}(\infty)$ (mOD)	$(0.3 \pm 0.4)\ 10^{-3}$	$(0.2 \pm 0.3)\ 10^{-3}$	$(0.5 \pm 0.3)\ 10^{-3}$	$(0.1 \pm 0.2)\ 10^{-3}$
K (s^{-4})	$(1.9 \pm 1.2)\ 10^{-14}$ (Global parameter)			$(2.5 \pm 0.8)\ 10^{-14}$

Table S3 Fitting parameters by interpolation of data in Figure 4B (last column) and Figure S9 with eq. S1. Data in Figure 4B are obtained averaging over the range 580 to 600nm.

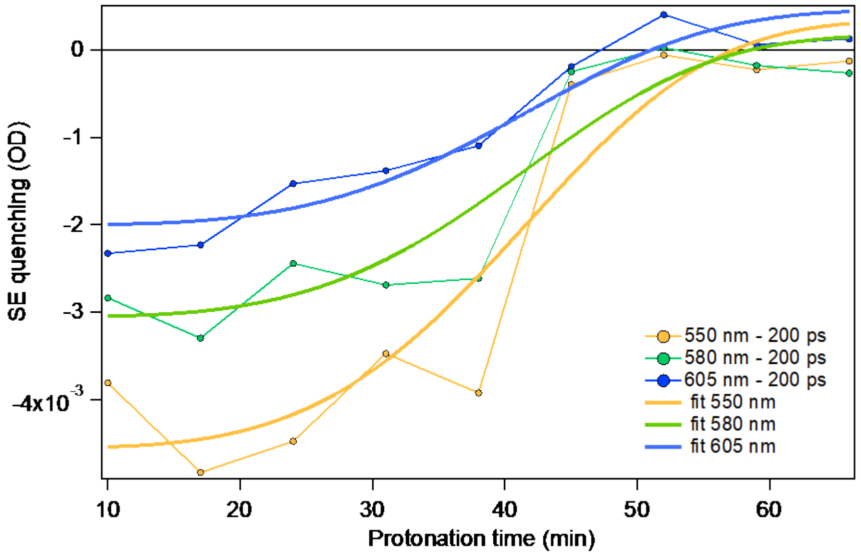

Figure S9. SE quenching kinetics monitored as the amplitude of TA signal at 220 ps (the longest scanned delay time) at different probe wavelengths. The solid line is obtained by fitting the data to eq. 1

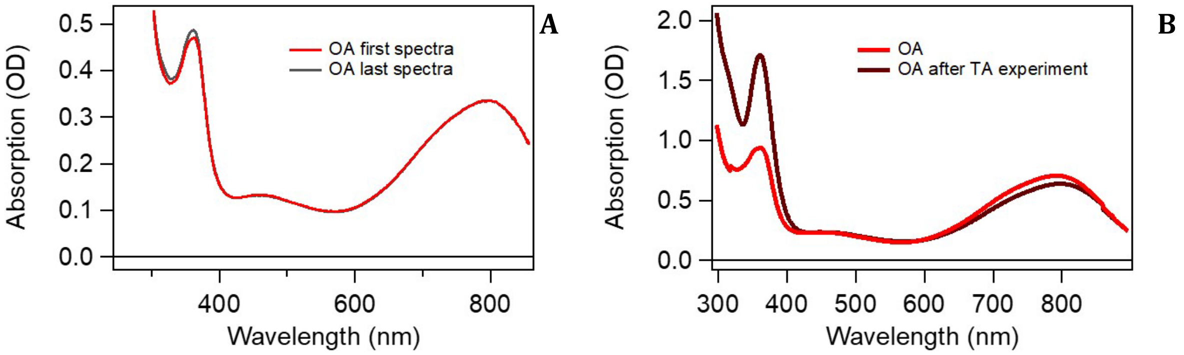

Figure S10. A) First (red) and last (black) steady state electronic absorption spectra measured with the sample kept in the spectrophotometer (mainly in the dark without exposition to the 400nm laser pump) in 2 hours of scans every 4 minutes. B) Steady state electronic absorption spectra before (red) and after (brown) the experiment. The increase of the signal at shorter wavelengths is tentatively assigned to the aggregate formation.

SI.7 Calculation of the fraction of excited molecules

As reported in the main text the experiment to investigate the photo-accumulation kinetic (Figure 4 and Figure S9) was carried out on 2 mL of solution with concentration c=4.43x10^{-4} M and with pulses of 65nJ energy each. Each scan was 7 min long with an excitation repetition rate of 500 Hz. According to these values the number of molecules in solution (N_{mol}) is

$$N_{mol}=N_A \bullet 4.43\ 10^{-4} \bullet 2\ 10^{-3} = N_A \bullet 9\ 10^{-7} = 6\ 10^{23} \bullet 9\ 10^{-7} = 0.54\ 10^{18},$$

while the total number of photons per pulse ($N_{ph/pulse}$) is

$$N_{ph/pulse} = 65\text{nJ}/h\nu_{400} = 1.3\ 10^{11}\ \text{photons/pulse}.$$

where $h\nu_{400}$ is the energy of a 400 nm photon (=3.1eV or 0.50 10^{-18} J).

Since the concentration was chosen to be roughly 0.3 OD at 400 nm and we took care to work in a linear regime the number of excited molecule per pulse ($N_{Exc\_mol/pulse}$) is equal to the number of absorbed photons:

$$N_{Exc\_mol/pulse} = N_{ph/pulse} \bullet 10^{-OD} = N_{ph/pulse} \bullet 0.5 = 6.5\ 10^{10}$$

and the fraction of excited molecule per pulse (R_{pulse}) which is given by

$$R_{pulse} = N_{Exc\_mol/pulse}/N_{mol} = 6.5/0.54\ 10^{-8} = 1.2\ 10^{-7}.$$

If we do not take care of reabsorption, the fraction of excited molecules after 40 minutes (R_{40min}) at 500 Hz of pump repetition rate is given by $R_{pulse} \bullet 40 \bullet 60 \bullet 500$= 14.4%, as reported in the main text. A more precise calculation should consider multiple excitations and R_{40min} can be calculated with the following formula:

$$R_{40min} = R_{pulse} \sum_{n=0}^{N_{ph}} \left(1 - R_{pulse}\right)^{n} = R_{pulse} \left[\frac{1-\left(1-R_{pulse}\right)^{N_{ph}+1}}{1-\left(1-R_{pulse}\right)}\right] = 13\%$$

where N_{ph} is the total number of pulses after 40 minutes, *i.e.* N_{ph}=40•60•500=1.2 10^6.

SI.8 Preliminary Results from calculations

As proved by the richness and complexity of the result herein reported as well as in ref. [11], a thoughtful and complete investigation with a non-adiabatic approach is mandatory to identify the possible conformers, the relaxation paths from the different excited states and the role of external parameters, as the solvent and the specific acid.

This extensive computational study is ongoing but some preliminary results, relevant to this article, can already be drawn. In particular, as already reported, [11] the different conformers, that can be optically and thermally populated, are present also in the ground state. Therefore, we have been carrying out an extensive characterization of the ground state potential energy surface to map its relevant minima. Consistently with this and previous works, we used Gaussian 16[22], with Becke-three-parameters exchange functional, including the Lee-Yang-Parr correlation potential B3LYP [23] and the 6-31+G(d)[24] [25] basis set. For the Pt atom, the SDD[26] effective core potential was utilized. Including the PCM[27] implicit solvent model using acetonitrile as a solvent. Conversely, the values of spin-orbit couplings (SOCs) and non-adiabatic coupling matrix elements (NACMEs), as well potential energy surfaces (PESs) and natural transition orbitals (NTOs) were calculated with the ORCA software package [28] using the B3LYP/G hybrid functional in combination with the def2-SVP basis set[29]. Relativistic effects were treated perturbatively using the zero-order regular approximation (ZORA)[30], with the uncontracted SARC-def2-SVP basis set[31] applied to the platinum atom, unless stated otherwise. In this section the computational states are labeled alphabetically to distinguish them from the experimentally measured states (namely $\boldsymbol{S_1}$ and $\boldsymbol{S_2}$)

The geometric scan along the dihedral angle describing the rotation of one of the flexible benzene units in the MBAdto ligand is shown in Figure S11. The α angle is defined as the rotation of the methyl group about the C–N bond. The global minimum is located at approximately 200°, while the lower of the two local minima occurs at 270°. The highest-energy minimum is found at around 70°. Variations of the α angle may be held responsible for driving the conformational changes in the system on longer time scale, leading to more accessible packing and aggregate formation. By comparing the PES of the system with and without HCl, we propose that the ejection event stabilizes the minimum at an α-torsion of 270°, reducing the overall barrier height and favoring its population.

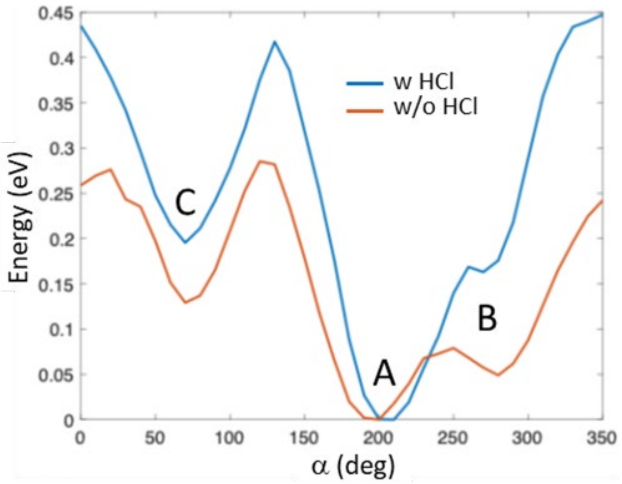

Figure S11. S_0 potential energy, with and without HCl (**1·HCl** and **1**, respectively), as a function of the dihedral angle between one of the methyl group the C–N bond (α-angle in the text). Three minima are observed and in the text are named in order of increasing energy, A, B and C. The used level of theory is[32] ωB97X-D3/def2-SVP.

Not surprisingly, we identified a local minimum which can be thermally populated from the global minimum because it is only 25 meV (40meV when calculated with ORCA) above the global minimum and it is separated by a barrier of 75 meV or less. Therefore, this configuration is the first candidate as the isomer responsible of the precipitate formation. These preliminary results are summarized in Figure S12, where the two configurations are compared. Relevant to this article, we infer from this figure two main results: 1) in the structure of the local minimum the two phen rings are more parallel and ordered, facilitating the formation of packed and ordered aggregates and ultimately of nanoparticles; 2) the local minimum shows an inversion of the electronic spatial distribution with respect to the global minimum, being the former centered on the quinoxdt and the latter on the MBAdto. As discussed in the main text, the latter can explain the observed blue-shift of the emission and the suppression of the ISC.

To estimate the barrier between the two configurations, we used linear morphing between the two structures to track a connecting path and to calculate the energy of the intermediate states. This approach only samples one possible transition, potentially overestimating the barrier, as lower-energy paths may exist.

To better describe the electronic (diabatic) nature of the possible excited-state pathways of interest, we report here the NTOs[33] in Figure S13, for the relevant minima identified in Figure S11. The relevant excited state (corresponding to S_2 in the main text) is named here as $S_{q/p}$ (see Table S4 for labelling). These are characterized by an electron and hole density both localized on the quinoxdt ligand.[11] The NTOs of state of S_1 (here named S_a) are also reported. We observe a substantial independence of the NTOs from the benzene units in the MBAdto ligand.

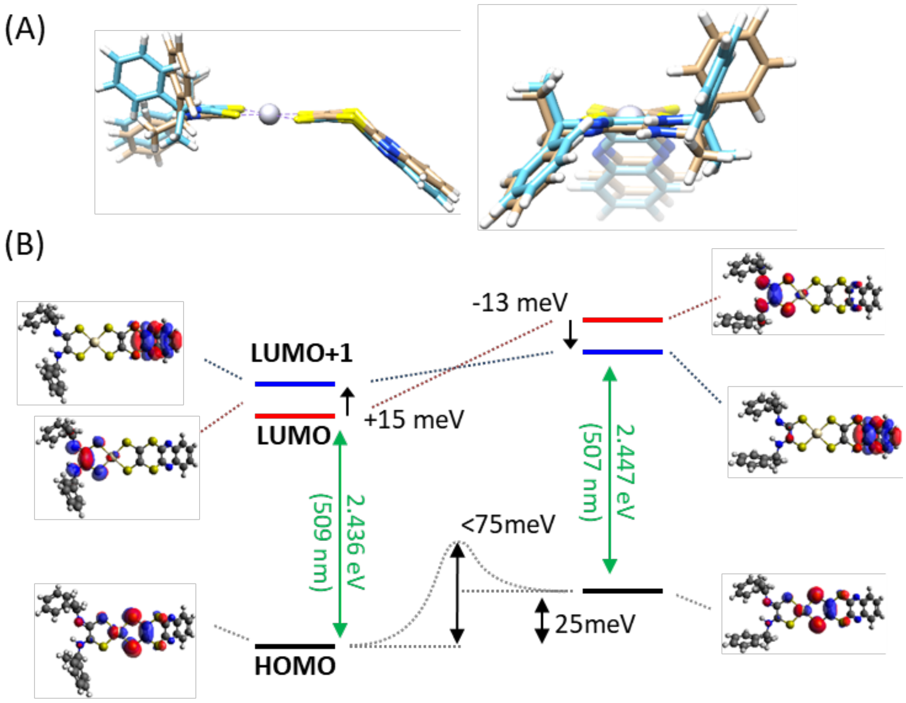

Figure S12. A) a comparison the equilibrium structures of the global and thermally accessible local minima (yellowish and blueish coloured, respectively). B) comparison of the energetics and of the molecular orbitals (only the HOMO and the first two LUMOs are shown) for the global (left) and local minima (right). The calculation are performed in acetonitrile at PCM level.

To estimate the coupling between states, the NACMEs are presented in Table S4 to Table S6. The reported values were calculated for the heteroleptic complex with HCl and represent the NACMEs between the ground state (GS) and the excited states (ES). The NACME values reported in Table S4 are computed for a single, static structure at minimum A. Notably, the coupling associated with $\boldsymbol{S}_{p/q}$ is relatively high compared to the nearby states. The NACME for the minimum B and C are shown in Table S5 and Table S6, respectively.

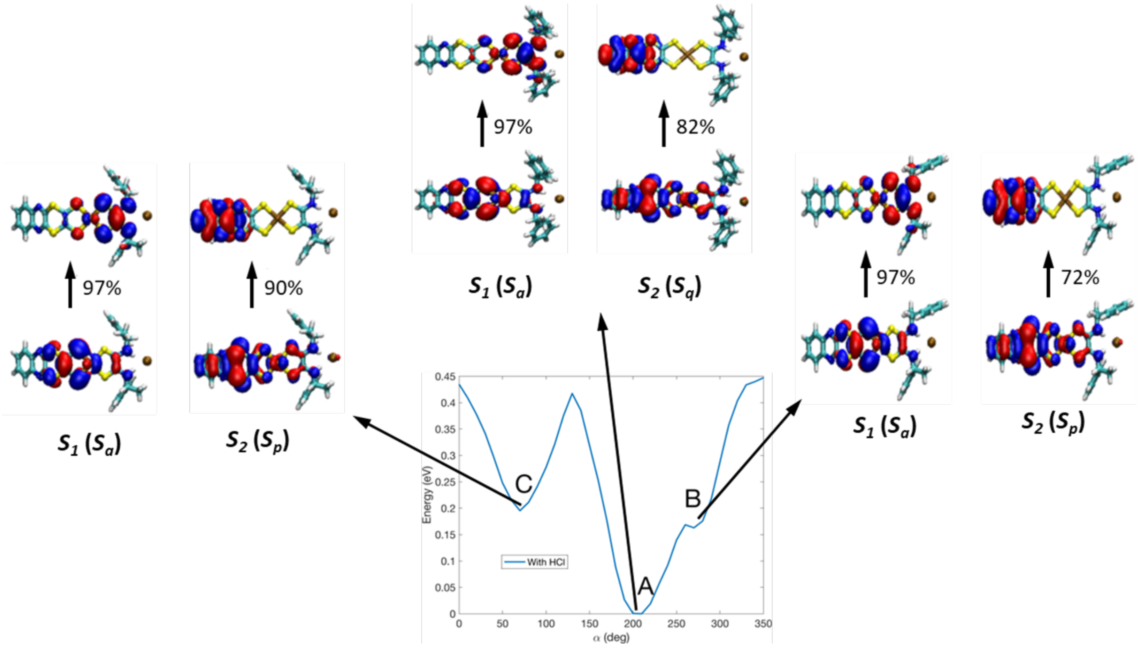

Figure S13: Natural transition orbitals (NTOs) from the ground state (S_0) for the minima of the adduct **1·HCl** shown in Figure S11. The displayed NTOs correspond to the lowest singlet excited state S_a (S_1) and to S_q (S_2), defined as the first higher excited single state with the NTO fully centered on the quinoxdt and with a not-negligible optical oscillator strength.

Table S4: (1st and 3rd columns) Nonadiabatic matrix element (NACMEs) between the ground state (GS) and the relevant singlet and triplet excited states (ES) (2nd and 3rd column, respectively) for the heteroleptic complex with HCl (**1·HCl**)**.** Data calculated for the optimized structure in the minimum A, coupling strength in $\boldsymbol{a_0}^{-1}$.

#	Singlet ES	$\left|\left\langle GS\left|\frac{d}{dx}\right|ES\right\rangle\right|$	Triplet ES	$\left|\left\langle GS\left|\frac{d}{dx}\right|ES\right\rangle\right|$
1	S_a (***S₁***)	0.97	T_b	1.18
2	S_b	0.31	T_c	1.00
3	S_c	0.56	T_d	0.14
4	S_d	0.23	T_e	0.19
5	S_e	0.24	T_f	0.21
6	S_f	0.36	T_g	0.30
7	S_g	0.089	T_h	0.28
8	S_h	0.16	T_i	0.27
9	S_i	0.18	T_j	0.13
10	S_j	0.20	T_k	0.20
11	S_k	0.24	T_l	0.54
12	S_l	0.16	T_m	0.20
13	S_m	0.24	T_n	0.21
14	S_n	0.19	T_o	0.12
15	S_o	0.21	T_p	0.41
16	S_p (***S₂***)	0.22	T_q	0.13
17	S_q	0.71	T_r	0.18
18	S_r	0.39	T_s	0.19
19			T_t	0.094
20			T_u	0.57
21			T_v	0.59
22			T_w	0.35

Table S5: the same data as in Table S4 but calculated for the optimized structure in the minimum B.

#	Singlet ES	$\left\|\left\langle GS\middle\|\frac{d}{dx}\middle\|ES\right\rangle\right\|$	Triplet ES	$\left\|\left\langle GS\middle\|\frac{d}{dx}\middle\|ES\right\rangle\right\|$
1	S_a (***S*1**)	0.90	T_b	0.15
2	S_b	0.27	T_c	0.97
3	S_c	0.55	T_d	0.34
4	S_d	0.22	T_e	1.1
5	S_e	0.23	T_f	0.19
6	S_f	0.35	T_g	0.18
7	S_g	0.097	T_h	0.33
8	S_h	0.16	T_i	0.35
9	S_i	0.13	T_j	0.62
10	S_j	0.22	T_k	0.40
11	S_k	0.19	T_l	1.0
12	S_l	0.19	T_m	0.052
13	S_m	0.25	T_n	0.36
14	S_n	0.24	T_o	0.25
15	S_o	0.38	T_p	0.63
16	S_p (***S*2**)	0.61	T_q	0.67
17			T_r	0.068
18			T_s	0.26
19			T_t	0.14
20			T_u	0.12

Table S6: the same data as in Table S4 but calculated for the optimized structure in the minimum C.

#	Singlet ES	$\left|\left\langle GS\left|\frac{d}{dx}\right|ES\right\rangle\right|$	Triplet ES	$\left|\left\langle GS\left|\frac{d}{dx}\right|ES\right\rangle\right|$
1	S_a ($\boldsymbol{S_1}$)	0.92	T_b	0.17
2	S_b	0.27	T_c	1.2
3	S_c	0.54	T_d	0.53
4	S_d	0.22	T_e	1.0
5	S_e	0.14	T_f	0.21
6	S_f	0.40	T_g	0.13
7	S_g	0.17	T_h	0.33
8	S_h	0.16	T_i	0.34
9	S_i	0.14	T_j	0.52
10	S_j	0.19	T_k	0.057
11	S_k	0.20	T_l	0.39
12	S_l	0.17	T_m	0.33
13	S_m	0.24	T_n	0.53
14	S_n	0.22	T_o	0.47
15	S_o	0.36	T_p	0.10
16	S_p ($\boldsymbol{S_2}$)	0.68	T_q	0.61
17			T_r	0.37
18			T_s	0.27
19			T_t	0.31
20			T_u	0.36
21			T_v	0.46

The SOC values for minima A, B and C of the acceptor are reported in Table S7, Table S8 and Table S9, respectively. We also calculated the SOC for **1** (data not shown), and we found that, except for a small increase of the average values, the variation and the dependence on the excited states show the same trend.

The experimentally photo-excited state S_q is identified based on the agreement with the excitation wavelength and the oscillator strength. The state T_w corresponds to the triplet state closest in energy to the singlet $\boldsymbol{S_q}$. The SOC between these two states is moderate mostly due to the limited participation of the Pt center to the electronic density reorganization, as discussed in the main text. However, since $\boldsymbol{S_q}$ lies close in energy to $\boldsymbol{T_w}$ (89 cm^{-1}), this energy barrier can be readily overcome, resulting in a high ISC rate. The SOC values in Table S8 between the $\boldsymbol{S_p}$ state and the $\boldsymbol{T_u}$ state are also of moderate magnitude. The same observation applies to the SOC values listed in Table S9 for the corresponding states.

Table S7: Spin-orbit coupling (SOC) between the first six lowest singlet and the closest triplet states and between the photo-excited singlet state (S_q) and the closest triplet state (T_w). The data ware calculated for the optimized static structure of the minimum A. The values are in cm^{-1}.

	T_a	T_b	T_c	T_d	T_e	T_w
S_0	108.2	504.1	19.0	1197.4	100.2	
S_a ($\boldsymbol{S_1}$)	17.0	400.0	32.2	362.8	38.5	
S_b	36.0	17.5	2.9	79.0	9.0	
S_c	706.6	16.5	15.0	102.7	712.4	
S_d	324.2	70.9	76.8	6.9	43.7	
S_e	1350.8	1843.3	25.3	114.1	1294.9	
S_q ($\boldsymbol{S_2}$)						36.8

Table S8: Same data as in Table S7 but calculated in the minimum B. T_u indicates the closest triplet state to the photoexcited singlet state S_q. The values are in cm^{-1}.

	T_a	T_b	T_c	T_d	T_e	T_u
S_0	82.6	486.5	23.6	1220.4	165.2	
S_a ($\boldsymbol{S_1}$)	35.8	439.2	35.0	353.6	99.2	
S_b	36.2	13.8	2.3	85.4	7.1	
S_c	718.0	15.0	11.7	92.5	660.5	
S_d	308.6	42.7	84.9	11.5	43.5	
S_e	1442.3	1842.9	20.7	79.2	1293.9	
S_p ($\boldsymbol{S_2}$)						45.1

Table S9: Same data as in Table S7 but calculated in the minimum C. T_v indicates the closest triplet state to the photoexcited singlet state Sq. The values are in cm^{-1}.

	T_a	T_b	T_c	T_d	T_e	T_v
S_0	63.5	493.9	22.0	1204.1	171.9	
S_a($\boldsymbol{S_1}$)	39.3	425.9	35.2	365.9	129.2	
S_b	36.8	13.2	2.4	84.0	5.8	
S_c	712.6	32.8	12.7	92.1	665.4	
S_d	318.7	29.0	85.9	7.6	48.6	
S_e	1526.9	1858.3	23.0	47.0	1383.4	
S_p ($\boldsymbol{S_2}$)						23.3

The SOC values reported in Table S7 to Table S9 allow, along with the corresponding single-triple state energy gap, to estimate roughly the ISC rate between the two states. As written in the main

text we observe that the energy gap between $\boldsymbol{S_2}$ ($\boldsymbol{S_{q/p}}$) and the closest triplet state ($\boldsymbol{T_{w/u/v}}$) in the adduct **1·HCl** is almost degenerate (<90cm^{-1}), facilitating ISC. Furthermore, we found that $\boldsymbol{S_2}$ ($\boldsymbol{S_{q/p}}$), which is responsible of the nK response, is very close to several triplet states (typically 2 to 4 triplet states within $\pm k_B T$ at room temperature or $\pm 210\ cm^{-1}$) allowing for a very efficient (sub-100 fs) ISC. Conversely, when we considered the lower excited states, we calculated that in the adduct, regardless the configuration, the $\boldsymbol{T_1}$ is 0.55eV (4700 cm^{-1}) lower than $\boldsymbol{S_1}$, whereas $\boldsymbol{T_2}$ is 0.75eV (6100 cm^{-1}) higher than $\boldsymbol{S_1}$. The relatively small value of the $\boldsymbol{S_1}$-$\boldsymbol{T_1}$ SOC and the important energy gap would suggest a rather long ISC, in agreement with our previous works.[11, 16]
In conclusion of this section, we want to comment on what is maybe the result most relevant to the article, namely that the second excited state ($\boldsymbol{S_b}$) shows systematically lower SOC values regardless of the triplet state. This speaks for a less efficient ISC from this state. Accordingly, to the model presented in the main article, this is the state that becomes the emissive, lowest excited state, after the ejection of the HCl. As can be seen from Figure S1C and D and from Figure S12, in this state, which corresponds to the HOMO→LUMO+1 transition, the excited electron density is completely segregated on the Quinoxdt ligand without any involvement of the Pt orbitals. This condition, which was tentatively identified in the main article as the cause of the dramatic decrease of the ISC rates after the HCl detachment, is now fully confirmed by the calculations.

SI.9 Global fit analysis of the traces from panel A of Figure 4

To quantify the time scales of the different processes responsible of the dynamics shown in Figure 4, we performed a global analysis of the traces in panel A in terms of a multiphasic exponential decay, where the time constants were considered as global variables, meaning the same for all the traces, and the amplitudes as local variables allowed to adapt to the respective curve. The outcome is reported in Figure S14.

All the traces are well described by six exponential decays, whose time constants and amplitudes as a function of HCl addition time are reported in panel B. The time constant of the last component is set to infinity to account for dynamics occurring on time scales much longer than herein investigated time window. It worse noticing that the acquisition time of each trace is 7 minutes (see the main text) and the reported addition time is the time when the scan is concluded. Therefore, the acquisition of the first trace (10min) was started 3 minutes after the HCl addittion and concluded 10 minutes later. As can also be inferred from Figure S14 and Figure S8C), this trace shows on the ps and sub-ps times (the time points first acquired) a signatures typical of **1**, whereas on the 100s ps time scales (the latest acquired spectra) it is much closer to the behaviour of the adducts (see trace at 17min). This means that this trace is due to a dynamical mixture of **1·HCl** and **1** . Accordingly, we should consider the second trace (17 min) more representative of the behaviour of the adducts. More relevant for the sake of this article, we should observe that rise of the stimulated emission is counted mainly by the fifth component of 69 ps, since its amplitude is always opposite in sign with respect to the long-lived component. We exclude that 23 ps, component describes a rise of the SE because. 1) it amplitude has the same sign of the long-lived component; 2) after quenching it does not go to zero, as the fifth one does; 3) the identical behaviour of the kinetics upon 800 nm excitation in this time range (see Figure S8C) proves that this component is not peculiar of the nK process. On the hand, all the components, including the 23 ps one, show a clear change following the quenching, which reveals that the molecules undergo a significant change upon aggregation. The fact that first four components are always present, on the contrary of the fifth and the sixth ones, suggest that they describe processes typical of the complex and not of the HCl dissociation.

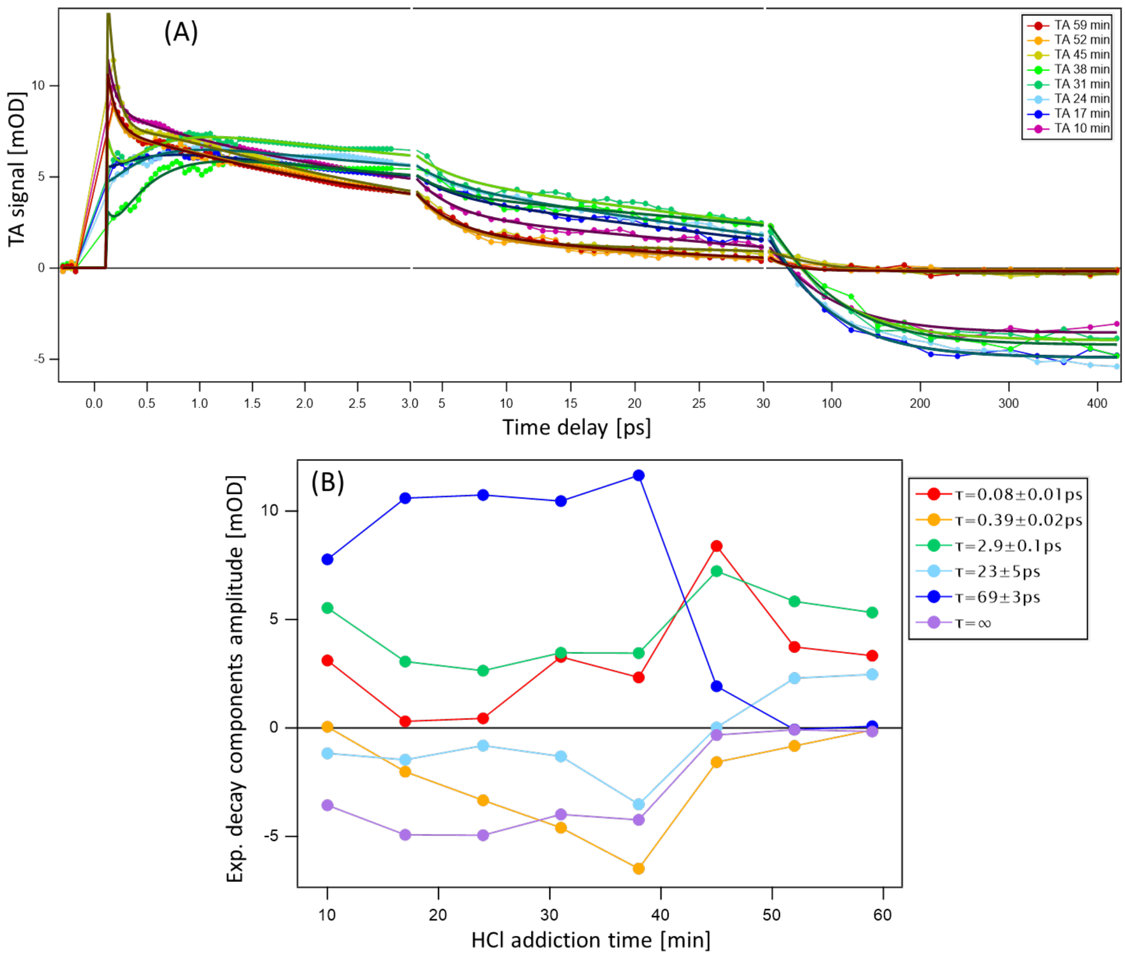

Figure S14. Kinetic traces from Figure 4A analysed with a global fitting approach in terms of 6 exponential decays: Best fitting curves and the parameters (amplitude and time constants) from the global fitting analysis are reported in panel A and B, respectively.

SI.10 Comment on the involvement of triplet states in the non-Kasha emission

As shortly discussed in the main text, we expect that on the tens-of-ps to ns timescale triplet states are populated due to heavy atom effects. This affirmation is also supported by the calculated the spin-orbit coupling reported in section SI.8 (Table S7 to Table S9, and relative discussion). allowing for a possible indirect mechanism of delayed fluorescence, where the emissive state is repopulated from triplet (dark) states.

It is worth bearing in mind that the amplitude of the stimulated emission (SE) is proportional to n, the population of the emissive state during the probe window, multiplied by k, the radiative rate. As discussed in the main text, to have a strong signal comparable to the ground state bealch (GSB), k must correspond to an allowed transition (i.e., 10^8–10^9 s^{-1}), and therefore n must be comparable to the number of excited molecules. Thus, even if the emissive state were populated indirectly, the back-transfer rate from the dark states should be sufficiently high to maintain a population comparable to the total excited-state population. In this regime, the overall lifetime is still determined by the emissive state itself: if its lifetime were only a few picoseconds, the total decay would also occur on the picosecond timescale. Nevertheless, invoking an indirect mechanism could allow for an intermediate scenario, with lifetimes of a few hundred picoseconds and back-transfer on the nanosecond scale. So even if we cannot exclude such a process, we can still conclude that our experimental results speak for an allowed transition with a long lifetime. About the exact nature of the back-transfer, it could be electronic, or it could be the ejection of the HCl itself.

References

(9) Attar, S.; Espa, D.; Artizzu, F.; Pilia, L.; Serpe, A.; Pizzotti, M.; Di Carlo, G.; Marchio, L.; Deplano, P. Optically Multiresponsive Heteroleptic Platinum Dithiolene Complex with Proton-Switchable Properties. *Inorg. Chem.* **2017**, *56* (12), 6763-6767.
(11) Gazzetto, M.; Artizzu, F.; Attar, S. S.; Marchio, L.; Pilia, L.; Rohwer, E. J.; Feurer, T.; Deplano, P.; Cannizzo, A. Anti-Kasha Conformational Photoisomerization of a Heteroleptic Dithiolene Metal Complex Revealed by Ultrafast Spectroscopy. *J. Phys. Chem. A* **2020**, *124* (51), 10687-10693.
(16) Frei, F.; Rondi, A.; Espa, D.; Mercuri, M. L.; Pilia, L.; Serpe, A.; Odeh, A.; Van Mourik, F.; Chergui, M.; Feurer, T.; et al. Ultrafast electronic and vibrational relaxations in mixed-ligand dithione-dithiolato Ni, Pd, and Pt complexes. *Dalton Trans.* **2014**, *43* (47), 17666-17676.
(18) Giannetto, A.; Puntoriero, F.; Barattucci, A.; Lanza, S.; Campagna, S. Tight-Contact Ion Pairs Involving Pt(II) Dithiooxamide Complexes: the Acid−Base Reactions between Hydrohalogenated Ion-Paired Complexes and Pyridine. *Inorg. Chem.* **2009**, *48* (21), 10397-10404. Giannetto, A.; Cordaro, M.; Campagna, S.; Lanza, S. Metal Complexes as Self-Indicating Titrants for Acid−Base Reactions in Chloroform. *Inorg. Chem.* **2018**, *57* (4), 2175-2183.
(19) Askari, B.; Rudbari, H. A.; Valente, A.; Bruno, G.; Micale, N.; Shivalingegowda, N.; Krishnappagowda, L. N. Synthesis, Characterization and Anticancer Studies of Rh(I), Rh(III), Pd(II) and Pt(II) Complexes Bearing A Dithiooxamide Ligand. *Chemistryselect* **2020**, *5* (2), 810-817.
(20) Jeffrey, G. A. *An introduction to hydrogen bonding*; Oxford University Press, 1997.
(21) Steiner, T. The Hydrogen Bond in the Solid State. *Angew. Chem. Int. Ed.* **2002**, *41* (1), 48-76.
(22) *Gaussian 16 Rev. C.01*; Wallingford, CT, 2016. (accessed.
(23) Becke, A. D. Density-functional thermochemistry. III. The role of exact exchange. *J. Chem. Phys.* **1993**, *98* (7), 5648-5652.
(24) Ditchfield, R.; Hehre, W. J.; Pople, J. A. Self-Consistent Molecular-Orbital Methods. IX. An Extended Gaussian-Type Basis for Molecular-Orbital Studies of Organic Molecules. *J. Chem. Phys.* **1971**, *54* (2), 724-728.
(25) Rassolov, V. A.; Ratner, M. A.; Pople, J. A.; Redfern, P. C.; Curtiss, L. A. 6-31G* basis set for third-row atoms. *J. Comput. Chem.* **2001**, *22* (9), 976-984.
(26) Fuentealba, P.; Preuss, H.; Stoll, H.; Von Szentpály, L. A proper account of core-polarization with pseudopotentials: single valence-electron alkali compounds. *Chem. Phys. Lett.* **1982**, *89* (5), 418-422. Schwerdtfeger, P.; Dolg, M.; Schwarz, W. H. E.; Bowmaker, G. A.; Boyd, P. D. W. Relativistic effects in gold chemistry. I. Diatomic gold compounds. *J. Chem. Phys.* **1989**, *91* (3), 1762-1774.
(27) Scalmani, G.; Frisch, M. J. Continuous surface charge polarizable continuum models of solvation. I. General formalism. *J. Chem. Phys.* **2010**, *132* (11), 114110.
(28) Neese, F. Software update: The ORCA program system—Version 5.0. *WIREs Computational Molecular Science* **2022**, *12* (5), e1606. Neese, F. The ORCA program system. *WIREs Computational Molecular Science* **2012**, *2* (1), 73-78.
(29) Schäfer, A.; Horn, H.; Ahlrichs, R. Fully optimized contracted Gaussian basis sets for atoms Li to Kr. *J. Chem. Phys.* **1992**, *97* (4), 2571-2577.
(30) van Lenthe, E.; Snijders, J. G.; Baerends, E. J. The zero-order regular approximation for relativistic effects: The effect of spin–orbit coupling in closed shell molecules. *J. Chem. Phys.* **1996**, *105* (15), 6505-6516.
(31) Pantazis, D. A.; Chen, X.-Y.; Landis, C. R.; Neese, F. All-Electron Scalar Relativistic Basis Sets for Third-Row Transition Metal Atoms. *J Chem Theory Comput* **2008**, *4* (6), 908-919.
(32) Lin, Y.-S.; Li, G.-D.; Mao, S.-P.; Chai, J.-D. Long-Range Corrected Hybrid Density Functionals with Improved Dispersion Corrections. *J Chem Theory Comput* **2013**, *9* (1), 263-272.
(33) Martin, R. L. Natural transition orbitals. *J. Chem. Phys.* **2003**, *118* (11), 4775-4777.
